# The SRSF1/circATP5B/miR-185-5p/HOXB5 feedback loop regulates the proliferation of glioma stem cells via the IL6-mediated JAK2/STAT3 signaling pathway

**DOI:** 10.1186/s13046-021-01931-9

**Published:** 2021-04-15

**Authors:** Junshuang Zhao, Yang Jiang, Haiying Zhang, Jinpeng Zhou, Lian Chen, Hao Li, Jinkun Xu, Guoqing Zhang, Zhitao Jing

**Affiliations:** 1 Department of Neurosurgery, the First Hospital of China Medical University, NO.155, North Nanjing Street, Heping District, Shenyang City, 110001 China; 2 Department of Neurosurgery, Shanghai Tenth People’s Hospital, Tongji University School of Medicine, Shanghai, 200072 People’s Republic of China; 3 International Education College, Liaoning University of Traditional Chinese Medicine, NO. 79 Chongshan East Road, Shenyang, 110042 China

**Keywords:** Glioma stem cells, CircATP5B, MiR-185-5P, HOXB5, SRSF1, IL6, JAK2/STAT3 signaling

## Abstract

**Background:**

Glioma is the most common and malignant tumor of central nervous system. The tumor initiation, self-renewal, and multi-lineage differentiation abilities of glioma stem cells (GSCs) are responsible for glioma proliferation and recurrence. Although circular RNAs (circRNAs) play vital roles in the progression of glioma, the detailed mechanisms remain unknown.

**Methods:**

qRT-PCR, western blotting, immunohistochemistry, and bioinformatic analysis were performed to detect the expression of circATP5B, miR-185-5p, HOXB5, and SRSF1. Patient-derived GSCs were established, and MTS, EDU, neurosphere formation, and limiting dilution assays were used to detect the proliferation of GSCs. RNA-binding protein immunoprecipitation, RNA pull-down, luciferase reporter assays, and chromatin immunoprecipitation assays were used to detect these molecules’ regulation mechanisms.

**Results:**

We found circATP5B expression was significantly upregulated in GSCs and promoted the proliferation of GSCs. Mechanistically, circATP5B acted as miR-185-5p sponge to upregulate HOXB5 expression. HOXB5 was overexpressed in glioma and transcriptionally regulated IL6 expression and promoted the proliferation of GSCs via JAK2/STAT3 signaling. Moreover, RNA binding protein SRSF1 could bind to and promote circATP5B expression and regulate the proliferation of GSCs, while HOXB5 also transcriptionally regulated SRSF1 expression.

**Conclusions:**

Our study identified the SRSF1/circATP5B/miR-185-5p/HOXB5 feedback loop in GSCs. This provides an effective biomarker for glioma diagnosis and prognostic evaluation.

**Supplementary Information:**

The online version contains supplementary material available at 10.1186/s13046-021-01931-9.

## Background

Glioma is the most common and malignant tumor among central nervous system cancers and is associated with poor prognosis in patients [1, 2]. Despite advances in surgery, radiotherapy, and chemotherapy, treatment outcomes in glioma patients remain poor, with an average survival time of approximately 15 months [3]. Glioma stem cells (GSCs) have been reported to be responsible for glioma and glioblastoma proliferation, therapeutic resistance, and recurrence due to their abilities in tumor initiation, self-renewal, and multi-lineage differentiation [4]. Therefore, studying the mechanisms of GSCs may provide important insights into potential strategies for glioblastoma therapy.

Circular RNA (circRNA) is an endogenous non-coding RNA mainly composed of exons and (or) introns that form a closed-loop structure through back splice sites between the 5′ and 3′ ends [5, 6]. Recently, circRNAs were proven to be involved in the cell proliferation, migration, invasion, apoptosis, and autophagy of several cancers [7–11]. Although several circRNAs have been reported to be involved in the biological functions of glioma, little is known about the function or molecular mechanisms of the novel circRNA, circATP5B, in glioma.

Homeobox (HOX) genes constitute a cluster of transcription factors with vital regulatory roles in embryonic development, cell differentiation, and tumorigenesis [12, 13]. Recent studies confirmed that HOXB5, a member of the homeobox gene family, is overexpressed and promotes cell proliferation and migration in various cancers, including breast, lung, and gastric carcinoma [14–17]. However, whether HOXB5 regulates the proliferation of glioma and its specific mechanism in the proliferation of GSCs remains unclear.

CircRNAs act their molecular function via several mechanisms, including miRNA sponging, binding to RNA-binding proteins, modulating translation, and miRNA sponging is the most common role of circRNAs in the development of tumors [18–20]. MiR-185-5p has been confirmed to be downregulated in glioma and acts as a suppressor gene in several biological processes involved in glioma pathogenesis [21, 22]. Our study found that circATP5B could regulate HOXB5 expression via miR-185-5p sponging in glioma.

Serine/arginine-rich splicing factor 1 (SRSF1) is an RBP that regulates RNA translation, transport, and nonsense-mediated RNA decay [23]. SRSF1 has also been identified as a potential oncogene that is overexpressed in several cancers including glioma [23–25]. It has been reported that circRNA acts as a sponge for the RBP in glioblastoma and whether SRSF1 can regulate circATP5B expression remains unclear.

In the present study, we firstly found that circATP5B and HOXB5 were overexpressed in glioma, especially in GSCs, and that circATP5B can upregulate HOXB5 expression via miR-185-5p sponging. Furthermore, gene set enrichment analysis (GSEA) showed that higher HOXB5 expression was associated with enrichment of IL6-mediated JAK2/STAT3 signaling according to the TCGA and CGGA datasets. Besides, HOXB5 can transcriptionally regulate the expression of IL-6, and SRSF1 can upregulate circATP5B expression. Therefore, our study found a novel feedback loop involving the SRSF1/circATP5B/miR-185-5p/HOXB5 axis that regulates the proliferation of GSCs and may provide a novel target for glioma therapy.

## Materials and methods

### Patient samples and ethical approval

Seventy clinical samples from glioma patients were collected from January 2007 to January 2012 at the First Affiliated Hospital of China Medical University. There were 20 samples of grade II, 25 samples of grade III, and 25 samples of grade IV glioma. Besides, adjacent brain tissues from ten of those patients were collected as normal control group. Clinical information including molecular subtypes of glioma such as IDH status, 1p/19q status, H3F3A status, and MGMT status for these samples is outlined in Table 1. This study had obtained the approval of the ethics committee of the First Affiliated Hospital of China Medical University, and every patient wrote informed consent.

**Table 1:** Relationship of circATP5B expression to clinical features of glioma patients

Clinical features		Samples (***n*** = 70)	CircATP5B expression^**a**^		***P*** value
			Low (***n*** = 35)	High (***n*** = 35)	
**Sex**	Male	38	18	20	*P* = 0.631
	Female	32	17	15	
**Age**	≤ 50	28	16	12	*P* = 0.329
	> 50	42	19	23	
**WHO grade**	II	20	15	5	***P*** **= 0.002**
	III	25	14	11	
	IV	25	6	19	
**IDH** **status**	Wild	33	9	24	***P*<0.001**
	Mutant	37	26	11	
**1p/19q status**	Codeletion	38	25	13	***P*** **= 0.004**
	Non-codeletion	32	10	22	
**H3F3A status**	Wild	39	24	15	***P*** **= 0.03**
	Mutant	31	11	20	
**MGMT** **status**	Methylation	42	26	16	***P*** **= 0.015**
	Unmethylation	28	9	19	

^a^ CircATP5B expression was detected by qRT-PCR and ranked from low to high. The high expression of circATP5B was defined as the expression level higher than the median expression level of circATP5B

### Cell culture and GSC isolation

Six patient-derived primary glioma stem cells from WHO grade II to IV (grade II: GSC201 and GSC203; grade III: GSC302 and GSC305; grade IV: GSC403 and GSC406) were isolated and neurosphere cultures were performed as previously described [26]. The detailed clinicopathological information is presented in Supplementary Table 1. In brief, freshly resected glioma samples were dissociated into single cells and cultivated in serum-free DMEM/F12 with 2% B27, 20 ng/mL rh-bFGF, and rh-EGF (Gibco, Gaithersburg, MD, USA). Western blotting was performed to detect the changes of CD133 and nestin in GSCs after culturing for day 0, day 1, day 2, day 3, day 4, and day 5. The stem cell markers of GSCs were detected by immunofluorescence staining of CD133 (#ab216323, Abcam Technology, Cambridge, UK) and nestin antibodies (#ab105389, Abcam), and the multi-lineage differentiation capacity of GSCs was detected by immunofluorescence staining of GFAP (#ab7260, Abcam) and βIII tubulin (#ab18207, Abcam). qRT-PCR was performed to detect the expression of circATP5B in glioma stem cells with different times of passage to ensure the stability of circATP5B expression in GSCs. The results showed that the expression of circATP5B did not change significantly within the 8th generation but decreased from the 10th generation to the 12th generation (Fig. S1j). Therefore, we chose cells less than 8 generations for the experiment. The human glioma cell line U87 was purchased from the Chinese Academy of Sciences cell bank (Shanghai, China), and was cultured in Dulbecco’s Modified Eagle’s Medium (DMEM; HyClone, Logan, UT, USA), supplemented with 10% fetal bovine serum (FBS; Gibco, Carlsbad, CA, USA) and 1% penicillin/streptomycin (Gibco) at 37 °C with 5% CO2.

### Lentiviral vector construction and transfection

The lentivirus transfection and efficacy measurements were performed as previously described [26]. The lentivirus-based vectors for circATP5B overexpression, HOXB5 overexpression, SRSF1 overexpression, RNAi-mediated knockdown of circATP5B, HOXB5 and SRSF1, and their negative controls were all constructed by Gene-Chem (Shanghai, China). The miR-185-5p mimic, inhibitor, and negative controls were obtained from Thermo Fisher Scientific (Assay ID: MH12486 and MC12486; Thermo Fisher Scientific, Waltham, MA, USA). The sequences of all siRNAs are listed in Supplementary Table 2. The transfection efficacy was detected by qRT-PCR and western blotting.

### RNA extraction, nuclear-cytoplasmic fraction, RNase R treatment and quantitative real-time PCR (qRT-PCR)

qRT-PCR was performed as previously described [26]. The total RNA of glioma tissues and GSCs were extracted via the Mini-BEST Universal RNA Extraction kit (TaKaRa, Kyoto, Japan) according to the manufacturer’s instructions. For circRNA and relative mRNA, the RNA was reverse transcribed into cDNA through a Prime Script RT Master Mix reagent kit (TaKaRa). For miRNA, cDNA was synthesized by the Prime Script™ RT reagent kit (TaKaRa, Shiga, Japan). Subsequently, the qRT-PCR assays were detected by the SYBR Green Master Mix (TaKaRa) with PCR LightCycler480 (Roche Diagnostics, Basel, Switzerland). The miR-185-5p expression was detected via the TaqMan Universal Master Mix II (Applied Biosystems, Foster.

City, CA, USA). Primers used in this study are listed in Supplementary Table 3. In addition, RNase R (Epicentre Technologies, Madison, USA) was used to confirm the existence of circATP5B and eliminate the effect of linear ATP5B RNA. GAPDH, U6 RNA, 5S RNA or β-actin were used as internal controls for circRNA, miRNA or mRNA. Moreover, Nuclear and cytoplasmic RNA fraction was isolated with PARIS™ Kit (Invitrogen, USA) according to the manufacturer’s instruction.

### Western blotting

Western blotting was performed as previously described [26]. In brief, a total cell protein extraction kit (KeyGen Biotechnology, Nanjing, China) was used to isolate the total proteins of glioma tissues or GSCs. Then, protein lysates were prepared, and the total protein for each sample was transferred onto the polyvinylidene difluoride (PVDF) membranes after SDS-polyacrylamide gel electrophoresis (SDS-PAGE), and blocked 2 h at room temperature with 2% bovine serum albumin (KeyGen Biotechnology). Subsequently, all these membranes were incubated overnight at 4 °C with the primary antibodies as below: HOXB5 (1:1000; #ab109375, Abcam), IL6 (1:1000; #ab233551, Abcam), p-JAK2 (1:500; #WL02997, Wanleibio, Shenyang, China), JAK2 (1:500; #ab195055, Abcam), p-STAT3 (1:1000; #WLP2412, Wanleibio), STAT3 (1:2000; #ab76315, Abcam), SRSF1 (1:500; #12929-2-AP, Proteintech, Rosemont, IL, USA) and β-actin (1:2000; #20536-1-AP, Proteintech). Following 2 h’ secondary antibodies (1:1000; #SA00001-2, Proteintech) incubation, all the bands were detected by a chemiluminescence ECL kit (Beyotime Biotechnology, Beijing, China) and quantified by Image J software (National Institutes of Health, Bethesda, MD, USA). The relative expression was calculated based on the internal control β-actin.

### Immunohistochemistry (IHC)

IHC was performed as previously described [26]. Firstly, the tumor tissues were embedded in paraffin, sliced into 4 mm sections, and labeled with primary antibodies as below: HOXB5 (1:100; #ab254882, Abcam), SRSF1 (1:100; #12929-2-AP, Proteintech), IL6 (1:100; #ab233551, Abcam), Ki67 (1:100; #ab92742, Abcam), CD133 (1:100; #ab216323, Abcam) and nestin (1:100; #ab105389, Abcam). The slices were then treated with an immunohistochemical labeling kit (MaxVision Biotechnology, Fuzhou, China) and imaged under a light microscope (Olympus, Tokyo, Japan). Finally, the staining intensity and the expression levels were evaluated according to the German immunohistochemical score [27].

### Immunofluorescence

Immunofluorescence staining was performed as previously described [27]. Firstly, the GSCs were fixed with 4% paraformaldehyde (solarbio, Beijing, China) for 10 min, permeabilized with 0.5% Triton X-100 (solarbio) for 20 min, blocked with 5% BSA (solarbio) for 1 h, and probed with primary antibodies as below: CD133 (#ab216323), nestin (#ab105389), GFAP (#ab7260), βIII-tubulin (#ab18207) (all 1:100; Abcam) at 4 °C overnight. Then, all the samples were treated with fluorescein isothiocyanate or rhodamine-conjugated secondary antibodies. Subsequently, the GSCs were counterstained with DAPI (Sigma, Shanghai, China) for 5 min. Finally, the staining was visualized by a laser scanning confocal microscope (Olympus).

### Enzyme-linked immunosorbent (ELISA)

ELISA was performed as previously described [27]. The Abcam’s IL-6 Human in vitro ELISA (Enzyme-Linked Immunosorbent Assay) kit (ab46027) was used to detect the concentrations of IL6 in the media supernatant of GSCs. All ELISA readings were normalized to the protein concentration in the control groups.

### Luciferase reporter assay

Luciferase reporter assays were performed as described previously [26]. Firstly, the luciferase reporter plasmids (circATP5B-wt and circATP5B-mt, HOXB5–3′-UTR-wt and HOXB5–3′-UTR-mt, IL6-wt and IL6-mt, and SRSF1-wt and SRSF1-mt) were constructed by Gene-Chem (Shanghai, China). The GSCs were then seeded into 96-well plates at a density of 5 × 10^3^ cells/well, transfected with luciferase reporter plasmids and performed other relative treatment for 48 h. Finally, the relative luciferase activities were detected via a Dual-Luciferase Reporter Assay System (Promega, USA). Relative luciferase activity was calculated as the ratio of firefly luciferase activity to Renilla luciferase activity. All experiments were independently repeated in triplicate.

### RNA immunoprecipitation (RIP) assay

The Imprint RNA Immunoprecipitation Kit (Sigma, USA) was used for RIP assay according to the manufacturer’s instructions. All GSCs under different conditions were lysed in RIP buffer including magnetic beads conjugated with negative control IgG, anti-AGO2, or anti-SRSF1 antibodies (Millipore, UK). After incubated with Proteinase K buffer (Omega, Shanghai, China), the immunoprecipitated RNAs were obtained. Finally, the qRT-PCR was performed to detect the precipitants.

### Chromatin immunoprecipitation (ChIP) assay

ChIP assays were performed via the ChIP Assay Kit (Beyotime Biotechnology, China) according to the manufacturer’s instructions. The chromatin complexes were immunoprecipitated via anti-HOXB5 antibody or normal rabbit IgG, and the purified DNA samples were detected by qRT-PCR. The primers are listed in Supplementary Table 3.

### RNA pull-down assay

The Pierce Magnetic RNA Protein pull-down Kit (Thermo Fisher Scientific) was performed to detect the interaction between circATP5B and SRSF1 according to the manufacturer’s instructions. Firstly, purified RNA was labeled by the biotinylated RNA probes, and then the positive control (input), negative control (antisense RNA), and biotinylated RNA were mixed and co-incubated with the proteins of GSCs at room temperature. The RNA-protein complex was added with magnetic beads to prepare a probe-magnetic bead complex. Finally, the complexes were detected by western blotting after being washed and boiled, and β-actin was used for the control.

### Cell viability assay

Cell viability assays were performed as described previously [26]. The GSCs were seeded into 96-well plates at a density of 1 × 10^3^ cells/well and incubated for 0, 24, 48, 72, 96, and 120 h. The cell viability was then detected via the CellTiter 96® Aqueous Non-Radioactive Cell Proliferation Assay Kit (Promega, Madison, WI, USA) according to the manufacturer’s instructions.

### EDU assay

EDU assays were performed as described previously [26]. According to the manufacturer’s instructions, the EDU assay was performed to detect the proliferation of GSCs by the EDU assay kit (Beyotime, Biotechnology, China). Firstly, the GSCs were seeded into 24-well plates at 1 × 10^5^ cells/well for 24 h, then 10 μM EDU reagent was added into the medium and incubated for 2 h. After being fixed with 4% paraformaldehyde (solarbio) and permeabilized with 0.5% Triton X-100 (solarbio), the GSCs were counterstained. Finally, the percentage of EDU positive cells was calculated via a laser scanning confocal microscope (Olympus).

### Neurosphere formation assay and in vitro limiting dilution assay

The neurosphere formation assay was performed as previously described [26]. Firstly, the GSCs were seeded into 24-well plates at a density of 200 cells/well and cultured in fresh medium for 7 days. Then, the relative neurosphere size was observed via a light microscope (Olympus). For in vitro limiting dilution assay, GSCs were seeded into 96-well plates at a gradient of 1, 10, 20, 30, 40 or 50 cells/well, and each gradient replicated 10 times. The number of neurospheres in each well was observed after 7 days incubation, and the neurosphere formation efficiency was calculated via the Extreme Limiting Dilution Analysis (http://bioinf.wehi.edu.au/software/elda) [28].

### Xenograft experiments

Xenograft experiments were performed according to the Animal Care Committee of China Medical University as described previously [26]. Firstly, the Six-week-old female BALB/c nude mice (Beijing Vital River Laboratory Animal Technology, Beijing, China) were divided into eight groups: the control, circATP5B-KD1, miR-185-5p-mimic, HOXB5-OE, SRSF1-OE, circATP5B-KD1 + HOXB5-OE, miR-185-5p-mimic+HOXB5-OE, SRSF1-OE + circATP5B-KD1 group. Each group with five mice were bred in the Laboratory Animal Center of China Medical University under specific pathogen-free conditions. The GSCs treated with different conditions were orthotopically injected into the mouse brain at 2 mm lateral and 2 mm anterior to the bregma by a stereotaxic apparatus (5 × 10^4^ cells per mouse). Each group was observed daily for distress or death signs, the mice were sacrificed and the whole-brain was isolated and sectioned coronally from most anterior to posterior. For each xenograft, the section with the largest tumor cross-sectional area was measured. Tumor volume was calculated according to the formula V=D × d^2^/2, where D represents the longest diameter and d represents the shortest diameter [29]. The overall survival time of survived mice was performed Kaplan-Meier survival analysis.

### Bioinformatics analysis

According to the expression of HOXB5 in the The Cancer Genome Atlas (TCGA, http://cancergenome.nih.gov) and the Chinese Glioma Genome Atlas (CGGA, http://www.cgga.org.cn), we ranked it from lower to higher. The value higher than the median is defined as the HOXB5 higher expression group, while the value lower than the median is defined as the HOXB5 lower expression group. Then, Gene set enrichment analysis (GSEA, http://www.broadinstitute.org/gsea/index.jsp) was used to analyze the enrichment of signaling pathways between the high and low HOXB5 expression. Four online databases, Starbase (http://starbase.sysu.edu.cn), TargetScan (www.targetscan.org), microRNA (http://www.microrna.org/microrna/home.do), and miRDB (http://mirdb.org) were used to predict possible miRNAs targeting HOXB5. Starbase and circBase (http://www.circbase.org/) databases were used to predict the potential circRNAs as sponges of miRNA and RBPs.

### Statistical analysis

All experiments were repeated at least three times, the results were expressed as the mean ± SD and the statistical analysis was performed by SPSS 22.0 software (SPSS, Chicago, IL, USA). The comparisons of two independent groups were detected by the chi-square test and two-tailed Student’s t-test. The statistical significance among three or more groups was evaluated by One-way analysis of variance. Pearson’s correlation analysis was used to detect the correlation between two groups. The Kaplan-Meier analysis and log-rank test were performed to analyze the survival rates of each group. Two-tailed *P* values < 0.05 were considered significant.

## Results

### CircATP5B upregulation in glioma correlates with poor patient survival

**Fig. 1:**
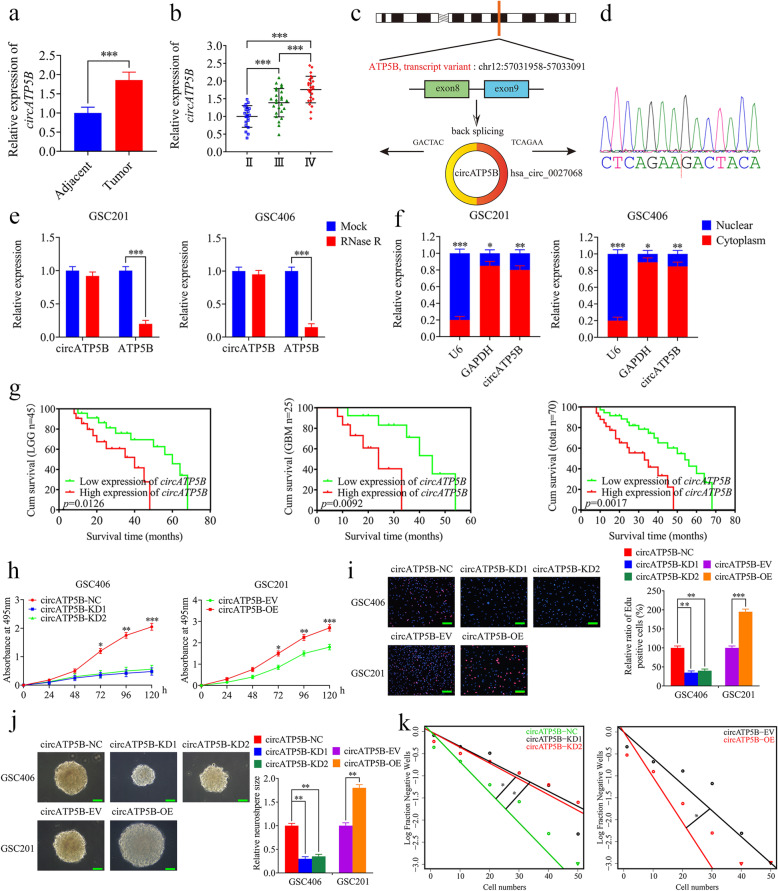
CircATP5B was upregulated in glioma and correlated with poor patient survival, and regulated the proliferation of GSCs. **a** CircATP5B was expressed at higher levels in glioma tissues, compared with adjacent brain tissues as detected by qRT-PCR. (Adjacent vs. Tumor: *n* = 10, *p* < 0.0001, Student’s t-test). **b** The expression level of circATP5B in different glioma tissues as detected by qRT-PCR. (grade II, *n* = 20; grade III, *n* = 25; grade IV, *n* = 25; III vs. II: *p* < 0.0001, IV vs. III: *p* < 0.0001, IV vs. II: *p* < 0.0001, One-Way ANOVA). **c** Schematic illustration of the formation of circATP5B via the circularization of exons in the ATP5B gene. **d** Sanger sequencing confirmed the head-to-tail splicing of circATP5B. **e** Relative expressions of circATP5B and ATP5B in both GSC406 and GSC201 were detected by qRT-PCR in the presence or absence of RNase R. (GSC201: *p* < 0.0001; GSC406: *p* < 0.0001; Student’s t-test). **f** circATP5B was mainly located in the cytoplasm by nuclear-cytoplasmic fractionation assay. (GSC201: U6: *p* < 0.0001, GAPDH: *p* = 0.026, circATP5B: *p* = 0.0057; GSC406: U6: *p* < 0.0001, GAPDH: *p* = 0.032, circATP5B: *p* = 0.0082, Student’s t-test). **g** Kaplan-Meier analysis showed the prognostic significance of the 70 glioma patients, 45 LGG patients, and 25 GBM patients with high versus low circATP5B expression detected by qRT-PCR. (LGG: *p* = 0.0126; GBM: *p* = 0.0092; 70 cases: *p* = 0.0017; Log-rank test). **h** MTS assays showed that circATP5B knockdown or overexpression affected the cell viability of GSCs (GSC406: *p* < 0.0001; GSC201: *p* < 0.0001; One-Way ANOVA). **i** The EDU assays showed that circATP5B knockdown or overexpression affected the proliferation of GSCs. Scale bar = 100 μm. (GSC406: *p* < 0.01; GSC201: *p* < 0.001; One-Way ANOVA). **j** The neurospheres formation assays showed that circATP5B knockdown or overexpression affected the relative size of the neurospheres of GSCs. Scale bar = 20 μm. (GSC406: *p* < 0.01; GSC201: *p* < 0.01; One-Way ANOVA). **k** circATP5B knockdown or overexpression affected the neurosphere-forming capacity of GSCs as detected by limiting dilution assays (GSC406: *p* < 0.05; GSC201: *p* < 0.05; ELDA analysis; circles represent corresponding points, triangles mean the point is outside of the log fraction number wells). EV: empty vector, OE: overexpression, NC: negative control, KD: knockdown. All data were expressed as the mean ± SD (three independent experiments). *p < 0.05; **p < 0.01; ***p < 0.001

We first performed qRT-PCR on both glioma specimens and adjacent brain tissues, the results showed that the relative expression of circATP5B in glioma was higher than that in adjacent brain tissues (Fig. 1a). We further performed qRT-PCR on different WHO grade glioma and found that circATP5B was more highly expressed in higher WHO grade glioma (Fig. 1b). Meanwhile, we also found that there was a correlation of circATP5B expression with molecular subtypes of glioma. Briefly, the patients with IDH mutant type status, 1p/19q codeletion, H3F3A wild type status, or MGMT methylation showed lower circATP5B expression than the patients with IDH wild type status, 1p/19q non-codeletion, H3F3A mutant type status, or MGMT unmethylation (Table 1). CircATP5B, also named hsa_circ_0027068 according to the annotation of circBase (http://www.circbase.org/), was spliced from exons 8 and 9 of the *ATP5B* gene (chr12: 57031958–57033091) and formed a sense-overlapping circular transcript of 451 nt (Fig. 1c). Sanger sequencing certified the head-to-tail splicing of circATP5B (Fig. 1d). We then determined whether the head-to-tail splicing of circATP5B results from trans-splicing or genomic rearrangement. To certify the stability of circATP5B, both GSC406 and GSC201 were treated with RNase R, which is a processive 3′ to 5′ exoribonuclease. It was found that circATP5B resisted digestion by RNase R, but the linear form of ATP5B was readily digested (Fig. 1e). Moreover, the results of nuclear-cytoplasm separation illustrated that circATP5B was predominantly localized in the cytoplasm (Fig. 1f) and indicated that it might be an appropriate diagnostic or prognostic marker. In addition, Kaplan–Meier survival analyses showed that the median survival times of lower-grade glioma patients, glioblastoma multiforme (GBM) patients, or total glioma patients with higher expression of circATP5B were all shorter than those in patients with lower circATP5B expression (Fig. 1g).

We cultured six patient-derived primary GSCs and hematoxylin and eosin were used to stain patient-derived glioma tissues (Fig. S1a). Immunofluorescence staining confirmed the enrichment of stem cell markers, CD133 and nestin (Fig. S1b). We also confirmed the differentiation capacity of GSCs with differentiation markers, GFAP and βIII tubulin (Fig. S1c). We further performed western blotting to detect the changes of CD133 and nestin in GSCs after culturing for day 0, day 1, day 2, day 3, day 4, and day 5. The results showed there was no significant difference (Fig. S1d). Besides, qRT-PCR showed that the expression of circATP5B was highest in WHO grade IV GSCs (GSC403 and GSC406), followed by WHO grade III GSCs (GSC302 and GSC305) and was lowest in WHO grade II GSCs (GSC201 and GSC203) (Fig. S1e). We found that the expression level of circATP5B in GSC406 was the highest and was the lowest in GSC201. Taken together, these results confirmed that circATP5B is overexpressed in glioma and correlates with poor patient survival.

### CircATP5B regulates the proliferation of GSCs

To detect the functions of circATP5B in GSCs, we selected GSC406 and GSC201 for circATP5B silencing or overexpression. qRT-PCR was performed to detect the transfection efficiency (Fig. S2a, b). Then, we evaluated the effects of circATP5B on the proliferation of GSCs via MTS and EDU assays. All of the results showed that the cell viability and EDU-positive rates were decreased in circATP5B-silenced GSC406, while the opposite results were acquired in circATP5B-overexpressed GSC201 (Fig. 1h, i). Furthermore, the relative size of the neurospheres formed by GSC406 was significantly smaller than those of the control group following circATP5B knockdown, while the opposite result was obtained in GSC201 after circATP5B overexpression (Fig. 1j). Limiting dilution assays also showed that the neurosphere-forming capacity was decreased in circATP5B-silenced GSC406 but increased in circATP5B-overexpressed GSC201 (Fig. 1k). Together, these results confirmed that circATP5B plays a vital role in promoting the proliferation of GSCs.

### HOXB5 is overexpressed in glioma and correlates with poor patient survival

**Fig. 2:**
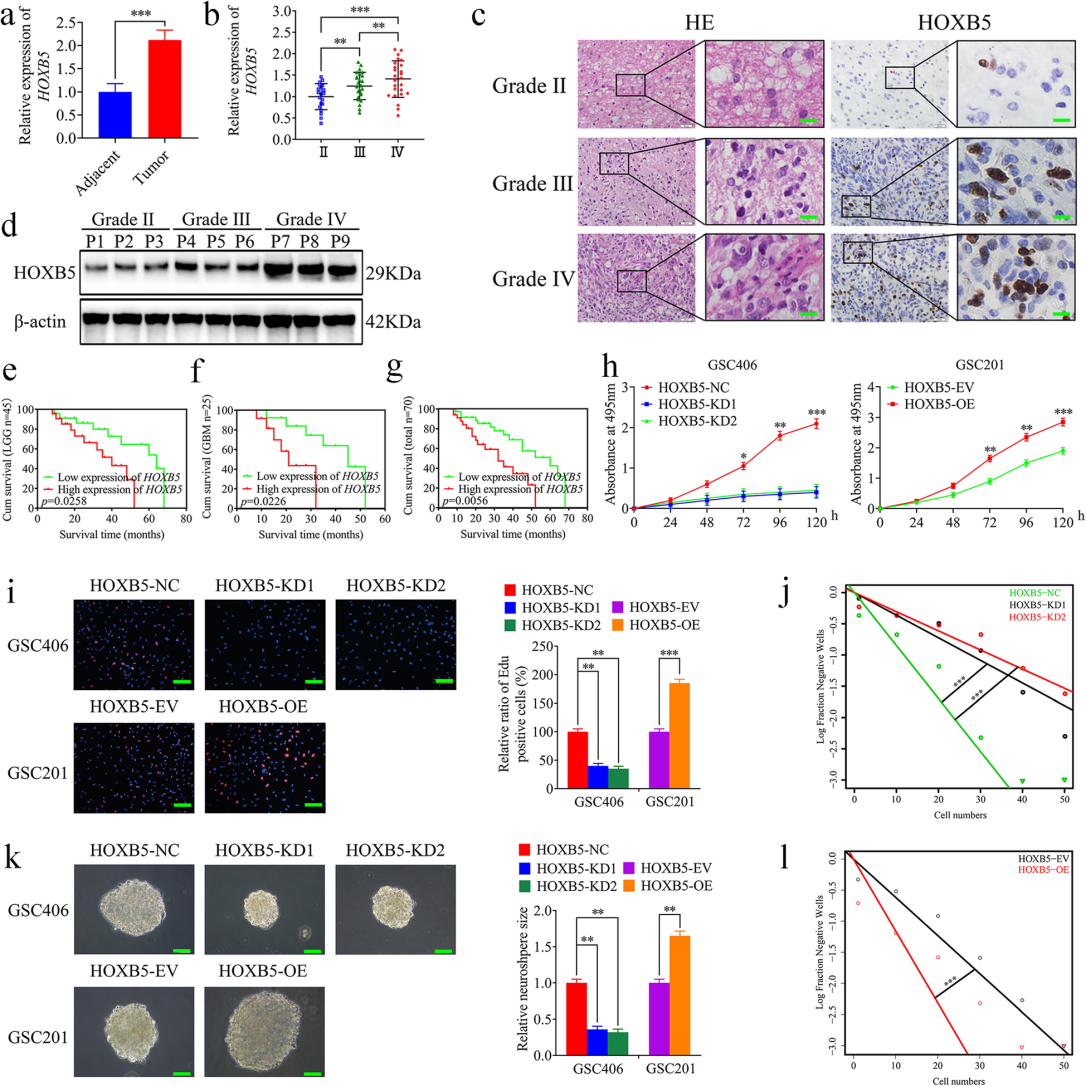
HOXB5 was overexpressed in glioma and correlated with poor patient survival, and regulated the proliferation of GSCs. **a** HOXB5 was expressed at higher levels in glioma tissues, compared with adjacent brain tissues as detected by qRT-PCR. (Adjacent vs. Tumor: *n* = 10, *p* < 0.0001, Student’s t-test). **b**-**d** The expression level of HOXB5 in different glioma tissues as detected by qRT-PCR (**b**), immunohistochemistry (**c**), and western blotting (**d**). Scale bar = 50 μm. (grade II, *n* = 20; grade III, *n* = 25; grade IV, *n* = 25; qRT-PCR: III vs. II: *p* < 0.01, IV vs. III: *p* < 0.01, IV vs. II: *p* < 0.0001, One-Way ANOVA; immunohistochemistry: III vs. II: *p* = 0.0042, IV vs. III: *p* = 0.0074, IV vs. II: *p* < 0.0001, One-Way ANOVA). **e**-**g** Kaplan-Meier analysis showed the prognostic significance of the 70 glioma patients, 45 LGG patients, and 25 GBM patients with high versus low HOXB5 expressions detected by qRT-PCR. (LGG: *p* = 0.0258; GBM: *p* = 0.0226; 70 cases: *p* = 0.0056; Log-rank test). **h** MTS assays showed that HOXB5 knockdown or overexpression affected the cell viability of GSCs. (GSC406: *p* < 0.0001; GSC201: *p* < 0.0001; One-Way ANOVA). **i** The EDU assays showed that HOXB5 knockdown or overexpression affected the proliferation of GSCs. Scale bar = 100 μm. (GSC406: *p* < 0.01; GSC201: *p* < 0.001; One-Way ANOVA). **k** The neurospheres formation assays showed that HOXB5 knockdown or overexpression affected the relative size of the neurospheres of GSCs. Scale bar = 20 μm. (GSC406: *p* < 0.01; GSC201: *p* < 0.01; One-Way ANOVA). **j** and **l** Limiting dilution assays showed that HOXB5 knockdown or overexpression affected the neurosphere-forming capacity of GSCs (GSC406: *p* < 0.001; GSC201: *p* < 0.001; ELDA analysis; circles represent corresponding points, triangles mean the point is outside of the log fraction number wells). EV: empty vector, OE: overexpression, NC: negative control, KD: knockdown. All data were expressed as the mean ± SD (three independent experiments). *p < 0.05; **p < 0.01; ***p < 0.001

HOXB5, as a member of the homeobox gene family, is a vital transcription factor, and HOXB5 overexpression is significantly correlated with cancer progression and a poor prognosis [30, 31]. However, the relationship between HOXB5 and glioma remains largely unknown. We found that the expression levels of HOXB5 in glioma tissues were higher than the adjacent brain tissues by qRT-PCR (Fig. 2a). Then, qRT-PCR, western blotting, and immunohistochemical analysis showed that HOXB5 expression was especially increased in higher glioma WHO grades (Fig. 2b-d). Moreover, we ranked the expression level from low to high according to the detection results of qRT-PCR. The value greater than the median is a higher expression, whereas the value less than the median is defined as a lower expression. Kaplan–Meier survival analyses showed that the median survival times of lower-grade glioma patients, GBM patients, or total glioma patients with higher HOXB5 expression were shorter than those for patients with lower HOXB5 expression (Fig. 2e-g). Both qRT-PCR and western blotting showed that HOXB5 was most highly expressed in WHO grade IV GSCs (GSC403 and GSC406), followed by WHO grade III GSCs (GSC302 and GSC305) and was lowest in WHO grade II GSCs (GSC201 and GSC203) (Fig. S1f, g). Furthermore, we found that the expression level of HOXB5 was higher in each type of GSC compared with other non-GSC types (Fig. S1h, i). Taken together, these results suggested that HOXB5 is overexpressed in glioma and associated with poor patient survival.

### HOXB5 regulates the proliferation of GSCs

To confirm whether HOXB5 correlated with the proliferation of glioma, we firstly performed qRT-PCR and western blotting to detect the efficiency of HOXB5 knockdown or overexpression (Fig. S2c–f). Then, we performed MTS and EDU assays and the results showed that cell viability and the rates of EDU-positive GSCs were decreased in HOXB5-silenced GSC406 but increased in HOXB5-overexpressed GSC201 (Fig. 2h, i). Moreover, the relative size of neurospheres formed by GSC406 was significantly smaller than those of the control group following HOXB5 knockdown, while the opposite result was obtained in HOXB5-overexpressed GSC201 (Fig. 2k). In addition, limiting dilution assays showed that the neurosphere-forming capacity was decreased in HOXB5-silenced GSC406, but increased in HOXB5-overexpressed GSC201 (Fig. 2j, l). In summary, our findings confirmed that HOXB5 is overexpressed in glioma and actively regulates the proliferation of GSCs.

### MiR-185-5p negatively regulates HOXB5 expression

**Fig. 3:**
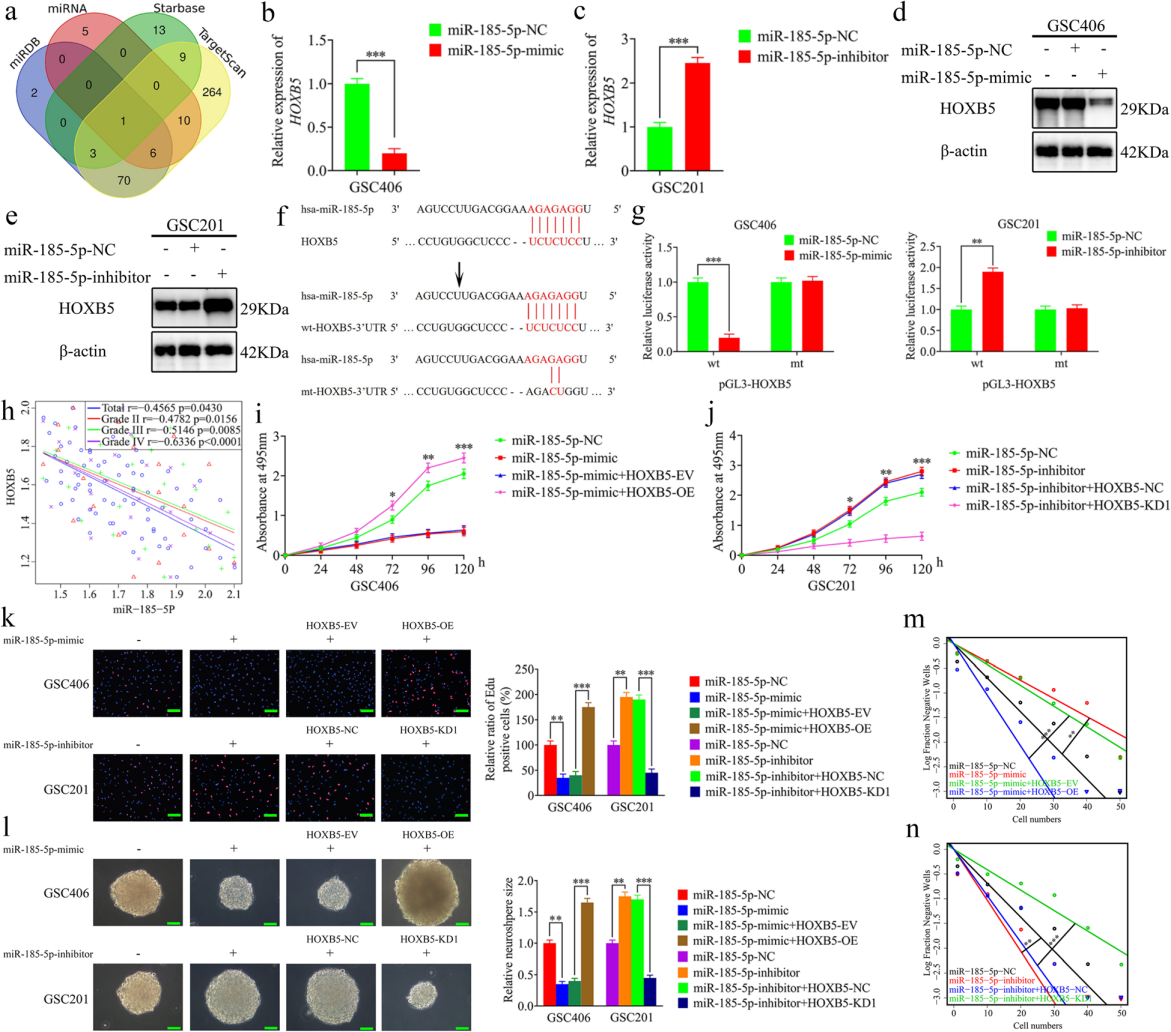
MiR-185-5p negatively regulated HOXB5 expression and suppressed the proliferation of GSCs. **a** Identification of a miRNA that potentially regulated HOXB5 expression based on microRNA, miRDB, Starbase, and TargetScan databases. **b, c** and **d, e** qRT-PCR (**b**, **c**) and western blotting (**d**, **e**) showed HOXB5 expression in GSCs after miR-185-5p mimic or inhibitor treatment. (GSC406: *p* < 0.0001; GSC201: *p* < 0.0001; Student’s t-test). **f** Schematic diagram of the putative miR-185-5p binding site in the 3′-UTR of HOXB5. **g** The luciferase reporter assays showed that miR-185-5p mimic or inhibitor affected the luciferase activities of HOXB5 in GSCs. (GSC406: *p* < 0.0001; GSC201: *p* = 0.0037; Student’s t-test). **h** The relative expression correlation between miR-185-5p and HOXB5 in 70 cases of glioma patients were detected by qRT-PCR. (Total: *r* = − 0.4565, *p* = 0.0430; Grade II: *r* = − 0.4782, *p* = 0.0156; Grade III: *r* = − 0.5146, *p* = 0.0085; Grade IV: *r* = − 0.6336, *p* < 0.0001; Pearson’s correlation analyses). **i** and **j** MTS assays showed that miR-185-5p mimic or inhibitor treatment affected the cell viability of GSCs and was reversed by HOXB5 overexpression or knockdown, respectively. (GSC406: *p* < 0.0001; GSC201: *p* < 0.0001; One-Way ANOVA). **k** The EDU assays showed that miR-185-5p mimic or inhibitor treatment affected the proliferation of GSCs and was reversed by HOXB5 overexpression or knockdown, respectively. Scale bar = 100 μm. (GSC406: *p* < 0.01; GSC201: *p* < 0.01; One-Way ANOVA). **l** The neurospheres formation assays showed that miR-185-5p mimic or inhibitor treatment affected the relative size of the neurospheres of GSCs and was reversed by HOXB5 overexpression or knockdown, respectively. Scale bar = 20 μm. (GSC406: *p* < 0.01; GSC201: *p* < 0.01; One-Way ANOVA). **m** and **n** Limiting dilution assays showed that miR-185-5p mimic or inhibitor treatment affected the neurosphere-forming capacity of GSCs and was reversed by HOXB5 overexpression or knockdown, respectively (GSC406: *p* < 0.01; GSC201: *p* < 0.01; ELDA analysis; circles represent corresponding points, triangles mean the point is outside of the log fraction number wells). EV: empty vector, OE: overexpression, NC: negative control, KD: knockdown. All data were expressed as the mean ± SD (three independent experiments). *p < 0.05; **p < 0.01; ***p < 0.001

We furtherly predicted that miR-185-5p was the only intersection bound to the 3′-UTR of HOXB5 according to microRNA, miRDB, TargetScan, and Starbase databases (Fig. 3a). We performed qRT-PCR and western blotting to confirm whether miR-185-5p regulated HOXB5 expression, and the results showed that HOXB5 expression levels were downregulated in miR-185-5p mimic-treated GSC406 but upregulated in miR-185-5p inhibitor-treated GSC201 (Fig. 3b-e). Pearson’s correlation analyses also confirmed a negative correlation between the expression levels of miR-185-5p and HOXB5 in each WHO grade glioma and in all glioma samples (Fig. 3h). Furthermore, we constructed luciferase reporter plasmids with wild-type and mutant forms of the HOXB5 3′-UTR (Fig. 3f), and luciferase reporter assays showed that the luciferase activity of HOXB5-wt vector was significantly decreased in miR-185-5p mimic-treated GSC406, while obviously increased in miR-185-5p inhibitor-treated GSC201. However, the luciferase activity of the HOXB5-mt vector showed no significant changes (Fig. 3g). Taken together, these results suggested that miR-185-5p negatively regulates HOXB5 expression through binding to the 3′-UTR of HOXB5.

### MiR-185-5p suppresses the proliferation of GSCs via HOXB5 inhibition

To confirm the effects of miR-185-5p and HOXB5 in the proliferation of GSCs, we performed rescue experiments. Both MTS and EDU assays showed that the cell viability and rates of EDU-positive GSCs were decreased in miR-185-5p mimic-treated GSC406, while these effects were reversed after HOXB5 overexpression. The opposite results were obtained in miR-185-5p inhibitor-treated GSC201, and the reverse was observed after HOXB5 knockdown (Fig. 3i-k). Furthermore, the relative size of neurospheres formed by GSC406 was significantly smaller than that of the control group after miR-185-5p mimic treatment, but became larger after HOXB5 overexpression. The opposite results were obtained in miR-185-5p inhibitor-treated GSC201, and this effect was reversed following HOXB5 knockdown (Fig. 3l). Limiting dilution assays showed that the neurosphere-forming capacity was decreased in miR-185-5p mimic-treated GSC406, but increased following HOXB5 overexpression. The opposite results were obtained in miR-185-5p inhibitor-treated GSC201, and the effect was reversed following HOXB5 knockdown (Fig. 3m, n). Together, miR-185-5p negatively regulated HOXB5 expression and suppressed the proliferation of GSCs.

### CircATP5B acts as a miRNA sponge of miR-185-5p

**Fig. 4:**
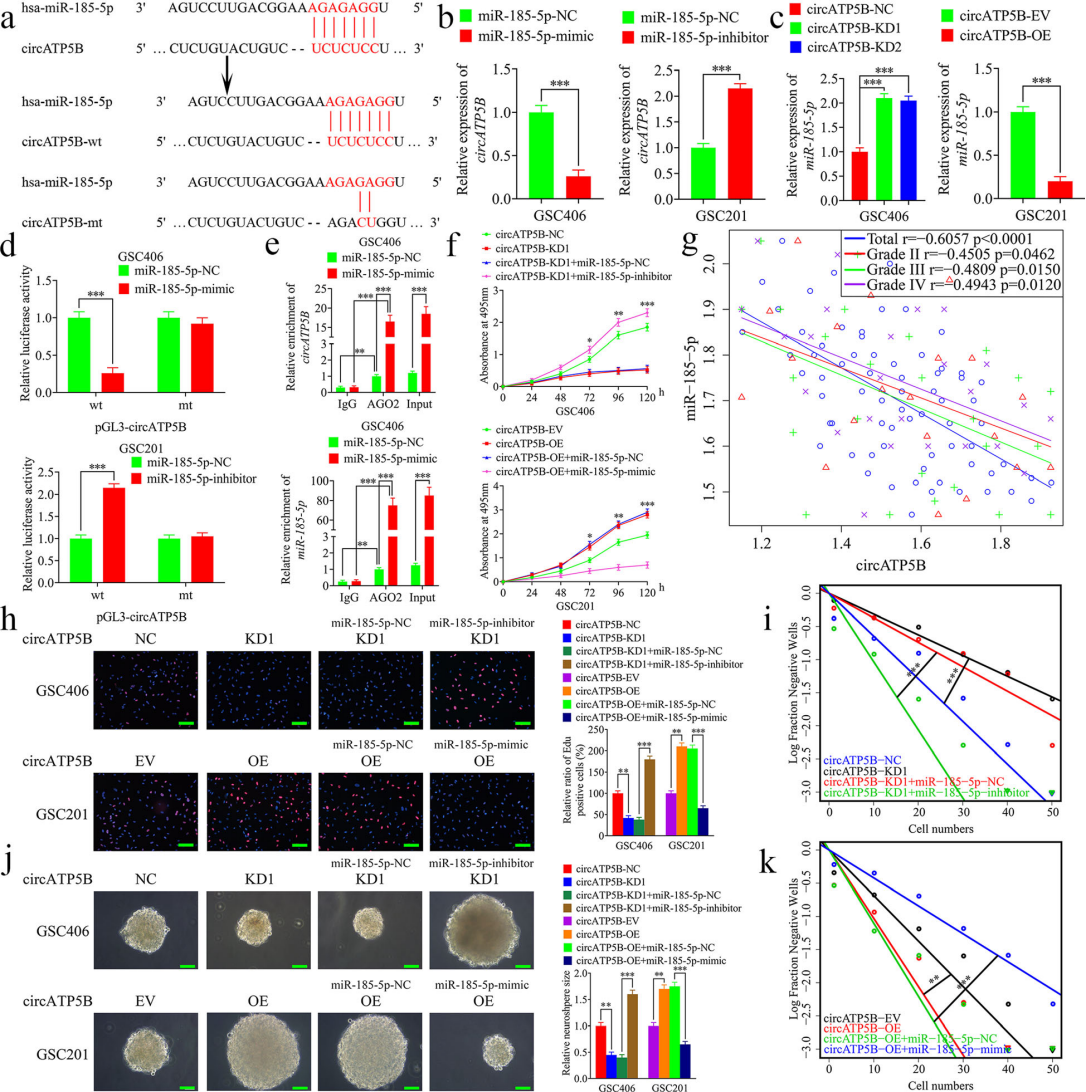
CircATP5B promoted the proliferation of GSCs through miRNA sponging of miR-185-5p. **a** Graphical illustration showing the predicted position of the circATP5B target on the miR-185-5p sequence. **b** and **c** qRT-PCR showed the relative expression of circATP5B in GSCs after miR-185-5p mimic or inhibitor treatment (**b**), and the relative expression of miR-185-5p in GSCs following circATP5B knockdown or overexpression (**c**). (**b**: GSC406: *p* < 0.0001; GSC201: *p* < 0.001; Student’s t-test; **c**: GSC406: *p* < 0.001; GSC201: *p* < 0.0001; One-Way ANOVA). **d** The luciferase reporter assays showed that miR-185-5p mimic or inhibitor affected the luciferase activities of circATP5B in GSCs. (GSC406: *p* < 0.0001; GSC201: *p* < 0.001; Student’s t-test). **e** The RNA immunoprecipitation (RIP) assay was performed in GSC406 after the miR-185-5p mimic or negative control was transfected, followed by qRT-PCR to detect the enrichment of circATP5B and miR-185-5p. (*p* < 0.01; One-Way ANOVA). **f** MTS assays showed that circATP5B knockdown or overexpression affected the cell viability of GSCs and was reversed by the miR-185-5p inhibitor or mimic treatment, respectively. (GSC406: *p* < 0.001; GSC201: *p* < 0.0001; One-Way ANOVA). **g** The relative expression correlation between circATP5B and miR-185-5p in 70 cases of glioma patients were detected by qRT-PCR. (Total: *r* = − 0.6057, *p* < 0.0001; Grade II: *r* = − 0.4505, *p* = 0.0462; Grade III: *r* = − 0.4809, *p* = 0.0150; Grade IV: *r* = − 0.4943, *p* = 0.0120; Pearson’s correlation analyses). **h** The EDU assays showed that circATP5B knockdown or overexpression affected the proliferation of GSCs and was reversed by miR-185-5p inhibitor or mimic treatment, respectively. Scale bar = 100 μm. (GSC406: *p* < 0.01; GSC201: *p* < 0.01; One-Way ANOVA). **j** The neurospheres formation assays showed that circATP5B knockdown or overexpression affected the relative size of the neurospheres of GSCs and was reversed by miR-185-5p inhibitor or mimic treatment, respectively. Scale bar = 20 μm. (GSC406: *p* < 0.01; GSC201: *p* < 0.01; One-Way ANOVA). **i** and **k** Limiting dilution assays showed that circATP5B knockdown or overexpression affected the neurosphere-forming capacity of GSCs and was reversed by miR-185-5p inhibitor or mimic treatment, respectively (GSC406: *p* < 0.001; GSC201: *p* < 0.01; ELDA analysis; circles represent corresponding points, triangles mean the point is outside of the log fraction number wells). EV: empty vector, OE: overexpression, NC: negative control, KD: knockdown. All data were expressed as the mean ± SD (three independent experiments). *p < 0.05; **p < 0.01; ***p < 0.001

CircRNAs have been confirmed to play crucial roles in several molecular mechanisms, such as miRNAs sponging, protein translation, and RNA-binding protein sponging. Increasing evidence has shown that miRNA sponging is the most common role of circRNAs in the development of tumors, including glioma [18, 32–34]. First, we predicted the potential target miRNAs of circATP5B according to Starbase and found that miR-185-5p possessed an accurate binding site for circATP5B (Fig. 4a). Second, qRT-PCR showed that the expression of circATP5B was decreased in miR-185-5p mimic-treated GSC406, but increased in miR-185-5p inhibitor-treated GSC201 (Fig. 4b). However, we found that the expression of miR-185-5p increased in circATP5B-silenced GSC406, but decreased in circATP5B-overexpressed GSC201 (Fig. 4c). To prove the above results in glioma cell lines, we repeated these experiments in U87 cells and obtained the same results (Fig. S3a-h). To confirm the possibility that miR-185-5p binds directly to circATP5B, we constructed luciferase reporter plasmids with wild-type and mutant circATP5B (Fig. 4a). The luciferase activity of circATP5B-wt vector significantly decreased in miR-185-5p mimic-treated GSC406, while obviously increased in miR-185-5p inhibitor-treated GSC201. However, the luciferase activity of the circATP5B-mt vector did not significantly change (Fig. 4d). Moreover, previous studies have shown that miRNAs bind to microRNA response elements (MREs) through RNA-induced silencing complex (RISC), an important component of which is AGO2 protein [35, 36]. Therefore, we performed an anti-AGO2 RIP assay to determine whether miR-185-5p and circATP5B were co-enriched in the RISC, and the results showed that both circATP5B and miR-185-5p were efficiently pulled down by anti-AGO2 antibody, compared with the IgG group. Furthermore, significant enrichment of circATP5B and miR-185-5p were observed after miR-185-5p mimic treatment, compared with the miR-185-5p negative control group (Fig. 4e). We also found a negative correlation between the expression levels of circATP5B and miR-185-5p in each WHO grade glioma and in all glioma samples via Pearson’s correlation analyses (Fig. 4g). In summary, these results demonstrated the direct interaction between circATP5B and miR-185-5p, and indicated that circATP5B may sponge miR-185-5p.

### CircATP5B promotes the proliferation of GSCs through miRNA sponging of miR-185-5p

To confirm the effects of circATP5B and miR-185-5p in the proliferation of GSCs, we performed rescue experiments. MTS and EDU assays showed that cell viability and the rates of EDU-positive GSCs were decreased in circATP5B-silenced GSC406, while this effect was reversed after miR-185-5p inhibitor treatment. However, the opposite results were obtained in circATP5B-overexpressed GSC201, and these upregulations were also reversed after miR-185-5p mimic treatment (Fig. 4f, h). These same results were also obtained in U87 cells (Fig. S3i-l). In addition, the relative size of the neurospheres formed by GSC406 was significantly smaller than those of the control group following circATP5B knockdown, but became larger after miR-185-5p inhibitor treatment. While the relative size of the neurospheres formed by GSC201 was obviously larger than that of the control group after circATP5B overexpression, and this reversed following miR-185-5p mimic treatment (Fig. 4j). Limiting dilution assays showed that the neurosphere-forming capacity was decreased in circATP5B-silenced GSC406, but increased following miR-185-5p inhibitor treatment. While the opposite results were acquired in circATP5B-overexpressed GSC201, and the increased neurosphere-forming capacity was reversed following miR-185-5p mimic treatment (Fig. 4i, k). Taken together, circATP5B promoted the proliferation of GSCs through sponging miR-185-5p, and there was a negative interaction between circATP5B and miR-185-5P.

### CircATP5B can upregulate the expression of HOXB5 through miRNA sponging of miR-185-5p

**Fig. 5:**
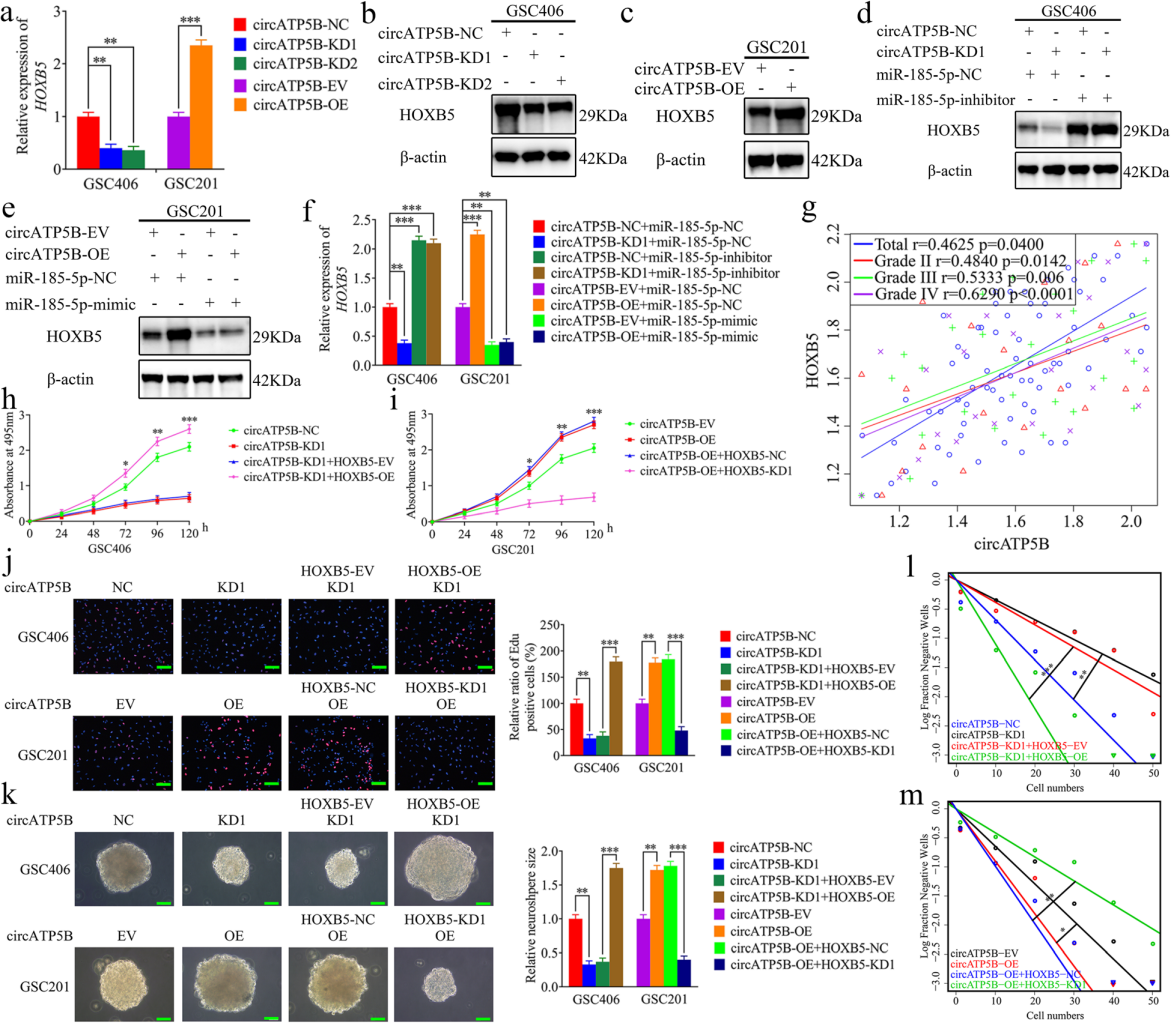
CircATP5B promoted the proliferation of GSCs by upregulating HOXB5 expression. **a**, **b**, **c** qRT-PCR (**a**) and western blotting (**b, c**) showed the expression of HOXB5 in GSCs after circATP5B knockdown or overexpression. (GSC406: *p* < 0.01; GSC201: *p* < 0.001; One-Way ANOVA). **d** The decreased expression of HOXB5 in GSC406 induced by circATP5B knockdown was reversed by miR-185-5p inhibitor treatment, as detected by western blotting. **e** The increased expression of HOXB5 in GSC201 induced by circATP5B overexpression was reversed by miR-185-5p mimic treatment, as determined by western blotting. **f** The effects of both circATP5B and miR-185-5p on the expression of HOXB5 in GSCs were detected by qRT-PCR. (GSC406: *p* < 0.01; GSC201: *p* < 0.01; One-Way ANOVA). **g** The relative expression correlation between circATP5B and HOXB5 in 70 cases of glioma patients were detected by qRT-PCR. (Total: *r* = 0.4625, *p* = 0.04; Grade II: *r* = 0.4840, *p* = 0.0142; Grade III: *r* = 0.5333, *p* = 0.006; Grade IV: *r* = 0.6290, *p* < 0.0001; Pearson’s correlation analyses). **h** and **i** MTS assays showed that circATP5B knockdown or overexpression affected the cell viability of GSCs and was reversed by HOXB5 overexpression or knockdown, respectively. (GSC406: *p* < 0.001; GSC201: *p* < 0.001; One-Way ANOVA). **j** The EDU assays showed that circATP5B knockdown or overexpression affected the proliferation of GSCs and was reversed by HOXB5 overexpression or knockdown, respectively. Scale bar = 100 μm. (GSC406: *p* < 0.01; GSC201: *p* < 0.01; One-Way ANOVA). **k** The neurospheres formation assays showed that circATP5B knockdown or overexpression affected the relative size of the neurospheres of GSCs and was reversed by HOXB5 overexpression or knockdown, respectively. Scale bar = 20 μm. (GSC406: *p* < 0.01; GSC201: *p* < 0.01; One-Way ANOVA). **l** and **m** Limiting dilution assays showed that circATP5B knockdown or overexpression affected the neurosphere-forming capacity of GSCs and was reversed by HOXB5 overexpression or knockdown, respectively (GSC406: *p* < 0.01; GSC201: *p* < 0.05; ELDA analysis; circles represent corresponding points, triangles mean the point is outside of the log fraction number wells). EV: empty vector, OE: overexpression, NC: negative control, KD: knockdown. All data were expressed as the mean ± SD (three independent experiments). *p < 0.05; **p < 0.01; ***p < 0.001

Since both circATP5B and HOXB5 had specific binding sites for miR-185-5p, to confirm whether circATP5B regulated HOXB5 expression via a miR-185-5p-mediated ceRNA mechanism in GSCs, we firstly detected the expression of HOXB5 via qRT-PCR and western blotting. The results showed that HOXB5 expression was downregulated in circATP5B-silenced GSC406, but upregulated in circATP5B-overexpressed GSC201 (Fig. 5a–c). In addition, we performed rescue experiments by additional treatment with miR-185-5p mimic or miR-185-5p inhibitor. Both the qRT-PCR and western blotting results showed that HOXB5 expression was increased in circATP5B-silenced GSC406 after miR-185-5p inhibitor treatment, while the expression of HOXB5 was decreased in circATP5B-overexpressed GSC201 after miR-185-5p mimic treatment (Fig. 5d-f). Moreover, we acquired the same results in U87 cells (Fig. S4a-h). Besides, Pearson’s correlation analyses among clinical glioma specimens showed strong positive correlations between circATP5B and HOXB5 expression in each WHO grade glioma and among the total glioma samples (Fig. 5g). In summary, circATP5B upregulated HOXB5 expression through sponging miR-185-5p.

### CircATP5B promotes the proliferation of GSCs by upregulating the expression of HOXB5

To confirm the effects of circATP5B and HOXB5 in the proliferation of GSCs, we performed rescue experiments. Both MTS and EDU assays showed that cell viability and the rates of EDU-positive GSCs were decreased in circATP5B-silenced GSC406, while these effects were reversed after HOXB5 overexpression. However, cell viability and the rates of EDU-positive GSCs were increased in circATP5B-overexpressed GSC201, and these effects were reversed after HOXB5 knockdown (Fig. 5h-j). Meanwhile, these same results were also obtained in U87 cells (Fig. S4i-l). Furthermore, the relative size of the neurospheres formed by GSC406 was significantly smaller than that of the control group after circATP5B knockdown, but became larger following HOXB5 overexpression. The opposite results were obtained in circATP5B-overexpressed GSC201, and were reversed after HOXB5 knockdown (Fig. 5k). Limiting dilution assays showed that the neurosphere-forming capacity was decreased in circATP5B-silenced GSC406, but increased following HOXB5 overexpression. The opposite results were obtained in circATP5B-overexpressed GSC201, but reversed after HOXB5 knockdown (Fig. 5l, m). Taken together, these results suggested that circATP5B actively regulates HOXB5 expression through a miR-185-5p-mediated ceRNA mechanism, and promotes the proliferation of GSCs by upregulating HOXB5 expression.

### HOXB5 transcriptionally regulates IL6 expression and activates JAK2/STAT3 signaling

**Fig. 6:**
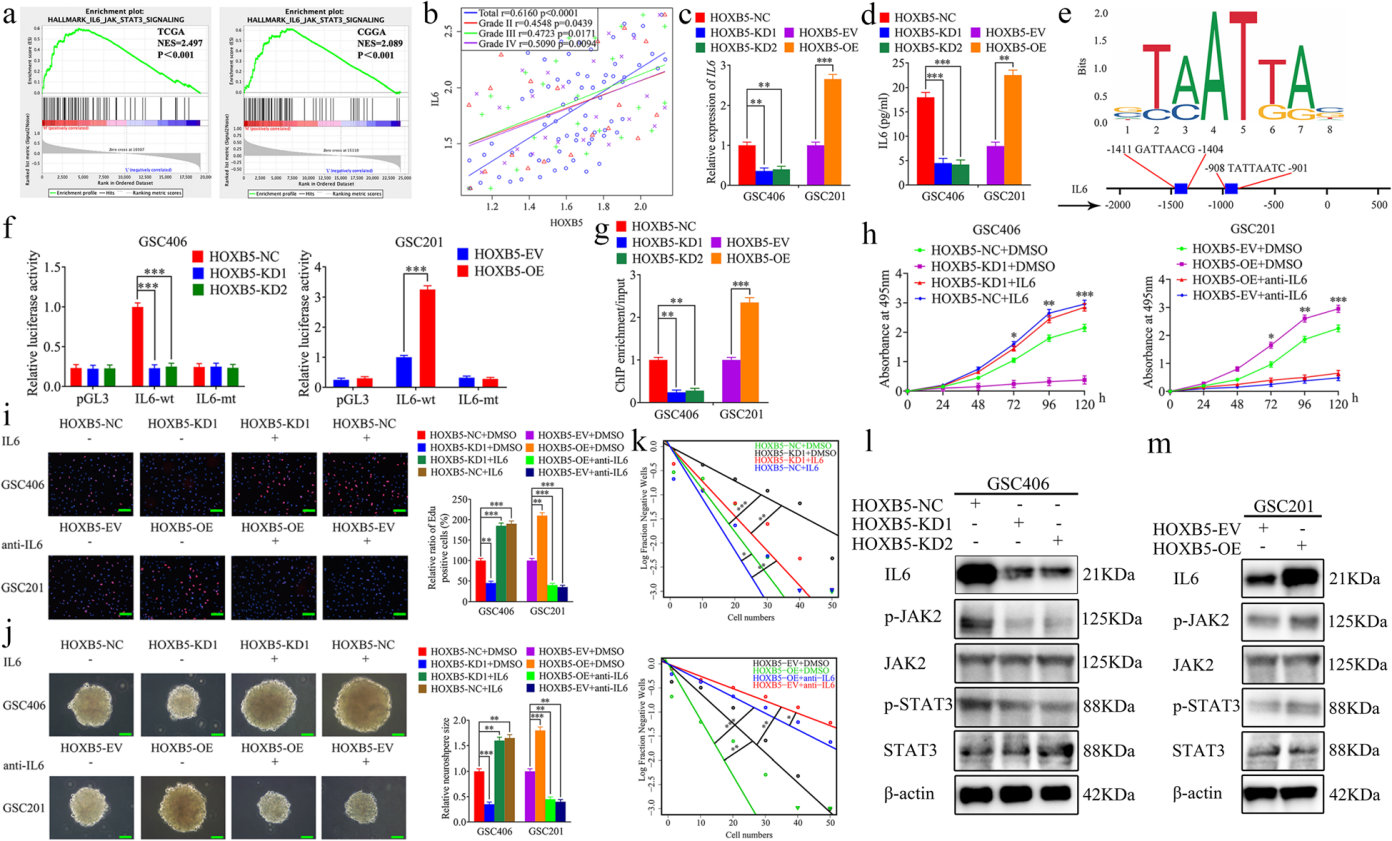
HOXB5 transcriptionally regulated IL6 expression and regulated the proliferation of GSCs via JAK2/STAT3 signaling. **a** TCGA and CGGA datasets showed that higher HOXB5 expression was associated with enrichment of IL6-mediated JAK2/STAT3 signaling. **b** The relative expression correlation between HOXB5 and IL6 in 70 cases of glioma patients was detected by qRT-PCR. (Total: *r* = 0.6160, *p* < 0.0001; Grade II: *r* = 0.4548, *p* = 0.0439; Grade III: *r* = 0.4723, *p* = 0.0171; Grade IV: *r* = 0.5090, *p* = 0.0094; Pearson’s correlation analyses). **c**, **d**, **l, m** qRT-PCR (**c**), ELISA (**d**), and western blotting (**l**, **m**) showed the IL6 expression was altered after HOXB5 knockdown or overexpression in GSCs. (**c**: GSC406: *p* < 0.01; GSC201: *p* < 0.001; One-Way ANOVA; **d**: GSC406: *p* < 0.001; GSC201: *p* < 0.01; One-Way ANOVA). **e** Sequence motif representing the consensus HOXB5 binding motif (JASPAR database), and Schematic diagram of the putative HOXB5 binding site in the 3′-UTR of IL6. **f** The luciferase reporter assays showed that HOXB5 knockdown or overexpression affected the luciferase activities of IL6 in GSCs. (GSC406: *p* < 0.001; GSC201: *p* < 0.001; One-Way ANOVA). **g** The ChIP qRT-PCR showed that HOXB5 bound to the promoter of IL6. (GSC406: *p* < 0.01; GSC201: *p* < 0.001; One-Way ANOVA). **h** MTS assays showed that HOXB5 knockdown or overexpression affected the cell viability of GSCs and was reversed by additional recombinant IL6 or anti-IL6, respectively. (GSC406: *p* < 0.001; GSC201: *p* < 0.001; One-Way ANOVA). **i** The EDU assays showed that HOXB5 knockdown or overexpression affected the proliferation of GSCs and was reversed by additional recombinant IL6 or anti-IL6, respectively. Scale bar = 100 μm. (GSC406: *p* < 0.01; GSC201: *p* < 0.01; One-Way ANOVA). **j** The neurospheres formation assays showed that HOXB5 knockdown or overexpression affected the relative size of the neurospheres of GSCs and was reversed by additional recombinant IL6 or anti-IL6, respectively. Scale bar = 20 μm. (GSC406: *p* < 0.01; GSC201: *p* < 0.01; One-Way ANOVA). **k** Limiting dilution assays showed that HOXB5 knockdown or overexpression affected the neurosphere-forming capacity of GSCs and was reversed by additional recombinant IL6 or anti-IL6, respectively. (GSC406: *p* < 0.05; GSC201: *p* < 0.05; ELDA analysis; circles represent corresponding points, triangles mean the point is outside of the log fraction number wells). **l** and **m** Western blotting showed the expression of downstream targets of the IL6-mediated JAK2/STAT3 signaling pathway with HOXB5 knockdown or overexpression in GSCs. EV: empty vector, OE: overexpression, NC: negative control, KD: knockdown. All data were expressed as the mean ± SD (three independent experiments). *p < 0.05; **p < 0.01; ***p < 0.001

To confirm the possible downstream mechanism of HOXB5 on glioma, we performed GSEA based on the expression of HOXB5. Both TCGA and CGGA datasets showed that higher HOXB5 expression was associated with enrichment of IL6-mediated JAK2/STAT3 signaling (Fig. 6a). Moreover, Pearson’s correlation analyses among clinical glioma specimens revealed significant positive correlations between HOXB5 and IL6 expression in each WHO grade glioma and among the total glioma samples (Fig. 6b). Then, qRT-PCR, western blotting, and ELISA assays showed that IL6 expression was downregulated in HOXB5-silenced GSC406, whereas IL6 expression was upregulated in HOXB5-overexpressed GSC201 (Fig. 6c, d, l, m). Besides, we further obtained the same results in U87 cells (Fig. S5a, b). Since HOXB5 is a transcription factor, we investigated whether HOXB5 transcriptionally regulated the expression of IL6 according to the Jaspar database (Fig. 6e). We performed luciferase reporter assays and found that the luciferase activity of the IL6-wt vector significantly decreased in HOXB5-silenced GSC406, while obviously increased in HOXB5-overexpressed GSC201. However, the luciferase activity of the IL6-mt vector showed no significant changes (Fig. 6f). ChIP assays also revealed that the enrichment of IL6 was decreased in GSC406 following HOXB5 knockdown, whereas it was increased in GSC201 after HOXB5 overexpression (Fig. 6g). In addition, we detected the downstream molecules of the *JAK2/STAT3* signaling pathway by western blotting and found that the expression levels of p-JAK2 and p-STAT3 were significantly downregulated in HOXB5-silenced GSC406, whereas the opposite results were obtained in HOXB5-overexpressed GSC201 (Fig. 6l, m). In summary, HOXB5 could transcriptionally regulate IL6 expression and activate JAK2/STAT3 signaling.

### HOXB5 regulates the proliferation of GSCs via IL6/JAK2/STAT3 signaling

We furtherly performed rescue experiments to confirm the effects of HOXB5 and IL6 in the proliferation of GSCs. Both MTS and EDU assays showed that cell viability and the rates of EDU-positive GSCs were decreased in HOXB5-silenced GSC406, while these effects were reversed following additional human recombinant IL6 treatment. The opposite results were obtained in HOXB5-overexpressed GSC201, and these effects were also reversed following additional IL6-neutralizing antibody treatment (Fig. 6h, i). Then, these same results were also acquired in U87 cells (Fig. S5c-e). In addition, the relative size of neurospheres formed by GSC406 was significantly smaller than that of the control group after HOXB5 knockdown, but became larger following additional human recombinant IL6 treatment. The opposite results were obtained in HOXB5-overexpressed GSC201, and the effects were reversed following additional IL6-neutralizing antibody treatment (Fig. 6j). Limiting dilution assays showed that the neurosphere-forming capacity was decreased in HOXB5-silenced GSC406, but increased after additional human recombinant IL6 treatment. The opposite results were obtained in HOXB5-overexpressed GSC201 and reversed following additional IL6-neutralizing antibody treatment (Fig. 6k). Taken together, HOXB5 transcriptionally regulated IL6 expression and promoted the proliferation of GSCs via JAK2/STAT3 signaling.

### SRSF1 can bind to and upregulate circATP5B expression

**Fig. 7:**
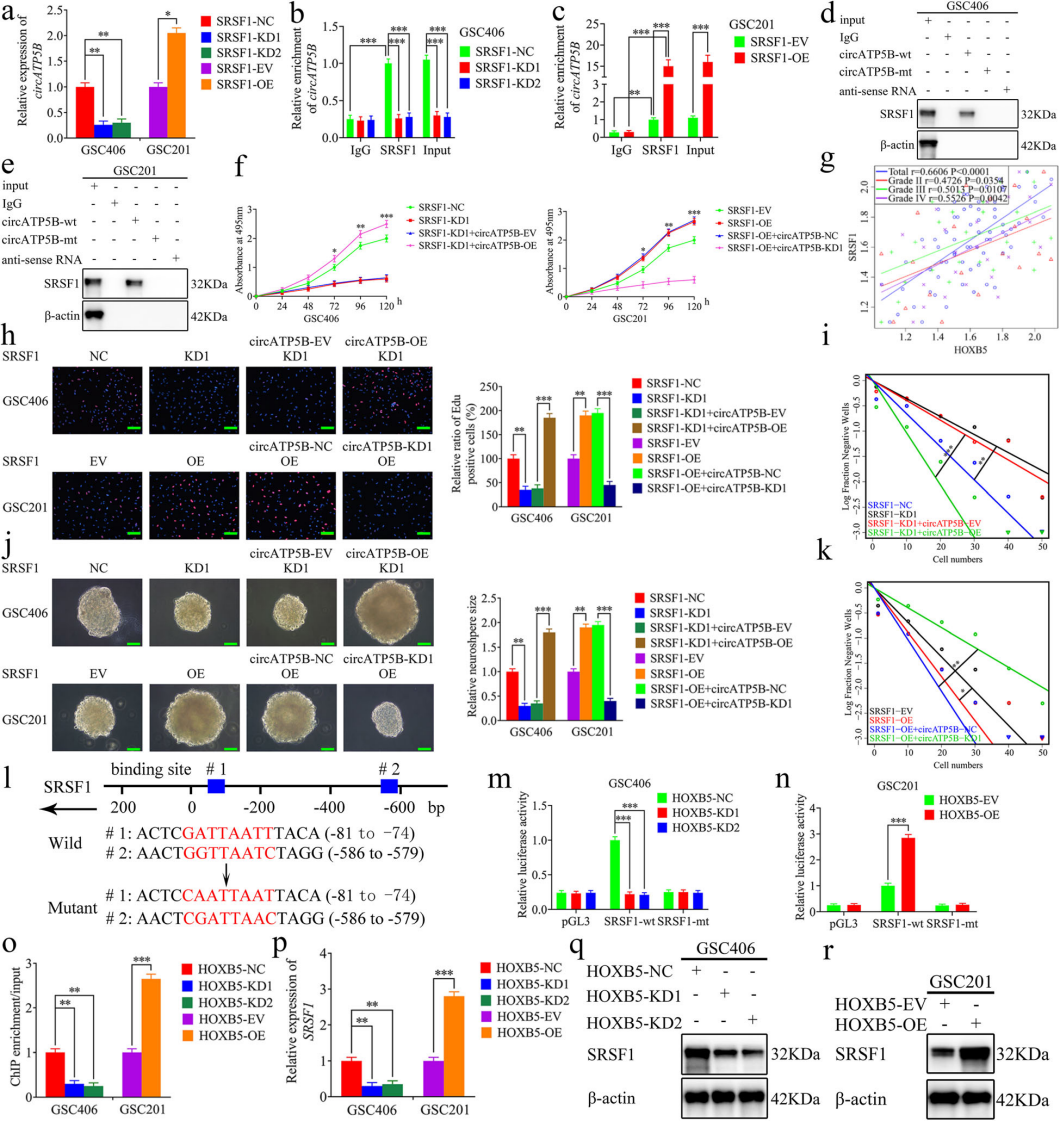
SRSF1 regulated the proliferation of GSCs by binding to circATP5B and upregulating circATP5B expression. **a** The relative expression of circATP5B after SRSF1 knockdown or overexpression was detected by qRT-PCR. (GSC406: *p* < 0.01; GSC201: *p* < 0.05; One-Way ANOVA). **b**, **c** The RIP assay was performed after SRSF1 knockdown (**b**) or overexpression (**c**), followed by qRT-PCR to detect the enrichment of circATP5B in GSCs. (GSC406: *p* < 0.001; GSC201: *p* < 0.01; One-Way ANOVA). **d**, **e** The RNA pull-down assays showed the SRSF1 protein immunoprecipitation with circATP5B as detected by western blotting. **f** MTS assays showed that SRSF1 knockdown or overexpression affected the cell viability of GSCs and was reversed by circATP5B overexpression or knockdown, respectively. (GSC406: *p* < 0.001; GSC201: *p* < 0.001; One-Way ANOVA). **g** The relative expression correlation between HOXB5 and SRSF1 in 70 cases of glioma patients were detected by qRT-PCR. (Total: *r* = 0.6606, *p* < 0.0001; Grade II: *r* = 0.4726, *p* = 0.0354; Grade III: *r* = 0.5013, *p* = 0.0107; Grade IV: *r* = 0.5526, *p* = 0.0042; Pearson’s correlation analyses). **h** The EDU assays showed that SRSF1 knockdown or overexpression affected the proliferation of GSCs and was reversed by circATP5B overexpression or knockdown, respectively. Scale bar = 100 μm. (GSC406: *p* < 0.01; GSC201: *p* < 0.01; One-Way ANOVA). **j** The neurospheres formation assays showed that SRSF1 knockdown or overexpression affected the relative size of the neurospheres of GSCs and was reversed by circATP5B overexpression or knockdown, respectively. Scale bar = 20 μm. (GSC406: *p* < 0.01; GSC201: *p* < 0.01; One-Way ANOVA). **i** and **k** Limiting dilution assays showed that SRSF1 knockdown or overexpression affected the neurosphere-forming capacity of GSCs and was reversed by circATP5B overexpression or knockdown, respectively (GSC406: *p* < 0.01; GSC201: *p* < 0.05; ELDA analysis; circles represent corresponding points, triangles mean the point is outside of the log fraction number wells). **l** Schematic diagram of the putative HOXB5 binding site in the 3′-UTR of SRSF1. **m** and **n** The luciferase reporter assays showed that HOXB5 knockdown or overexpression affected the luciferase activities of SRSF1 in GSCs. (GSC406: *p* < 0.001; GSC201: *p* < 0.001; One-Way ANOVA). **o** The ChIP qRT-PCR showed that HOXB5 bound to the promoter of SRSF1. (GSC406: *p* < 0.01; GSC201: *p* < 0.001; One-Way ANOVA). **p**, **q**, **r** qRT-PCR (**p**) and western blotting (**q, r**) showed the SRSF1 expression was affected after HOXB5 knockdown or overexpression in GSCs. (GSC406: *p* < 0.01; GSC201: *p* < 0.001; One-Way ANOVA). EV: empty vector, OE: overexpression, NC: negative control, KD: knockdown. All data were expressed as the mean ± SD (three independent experiments). *p < 0.05; **p < 0.01; ***p < 0.001

Splicing factor SRSF1 is a typical splicing factor protein that, in addition to its function in splicing, also plays a crucial role in nonsense-mediated mRNA decay, mRNA export, and translation [37]. Splicing is considered the main mechanism by which circRNAs originate, and SRSF1 is upregulated and functions as an oncoprotein in several cancers [38]. We found that SRSF1 was the most probable RBP to interact with circATP5B, according to the Starbase database with the highest “ClipExpNum”. We firstly selected GSC406 and GSC201 to perform SRSF1 knockdown and overexpression assays, and both qRT-PCR and western blotting were used to detect the efficiency of SRSF1 knockdown or overexpression (Fig. S2g–j). Then, qRT-PCR showed that circATP5B expression was downregulated in SRSF1-silenced GSC406, but upregulated in SRSF1-overexpressed GSC201 (Fig. 7a). Meanwhile, we obtained the same results in U87 cells (Fig. S6a, b). Furthermore, we performed a RIP assay to detect whether SRSF1 bound to circATP5B and found that the relative enrichment of circATP5B in the anti-SRSF1 group was significantly increased compared with the IgG-treated group. The relative enrichment of circATP5B in the anti-SRSF1 group was obviously decreased after SRSF1 knockdown, but increased after SRSF1 overexpression. However, the relative enrichment of circATP5B in the IgG-treated group showed no significant changes (Fig. 7b, c). Moreover, RNA pull-down assays showed that circATP5B-wt pulled down SRSF1 in GSC406 and GSC201, while this effect was not seen with circATP5B-mt (Fig. 7d, e). Together, these results suggested that, as an RBP and splicing factor, SRSF1 promotes the expression of circATP5B.

### SRSF1 regulates the proliferation of GSCs by upregulating circATP5B expression

To confirm the effects of SRSF1 and circATP5B in the proliferation of GSCs, MTS and EDU assays were performed. Both assays showed that cell viability and the rates of EDU-positive GSCs were decreased in SRSF1-silenced GSC406, and these effects were reversed following circATP5B overexpression. The opposite results were obtained in SRSF1-overexpressed GSC201, and the effects were also reversed following circATP5B knockdown (Fig. 7f, h). Besides, these same results were also obtained in U87 cells (Fig. S6c-e). In addition, the relative size of neurospheres formed by GSC406 was significantly smaller than that of the control group after SRSF1 knockdown, but became larger following circATP5B overexpression. The opposite results were obtained in SRSF1-overexpressed GSC201, and the effects were reversed following circATP5B knockdown (Fig. 7j). Limiting dilution assays showed that the neurosphere-forming capacity was decreased in SRSF1-silenced GSC406, but increased after circATP5B overexpression. While the opposite results were obtained in SRSF1-overexpressed GSC201, and these effects were reversed following circATP5B knockdown (Fig. 7i, k). Taken together, SRSF1 promoted the proliferation of GSCs by binding to and upregulating circATP5B expression.

### HOXB5 transcriptionally regulates SRSF1 expression in GSCs

Since HOXB5 is a transcription factor and SRSF1 is an RBP, we determined whether HOXB5 transcriptionally regulates SRSF1 expression in GSCs. We designed two binding sites for HOXB5 in the promoter of SRSF1 according to the Jaspar database (Fig. 7l), then performed luciferase reporter assays. The luciferase activity of SRSF1-wt vector was significantly decreased in HOXB5-silenced GSC406, while obviously increased in HOXB5-overexpressed GSC201. However, the luciferase activity of SRSF1-mt vector showed no obvious changes (Fig. 7m, n). In addition, Pearson’s correlation analyses among clinical glioma specimens showed significant positive correlations between HOXB5 and SRSF1 expression in each WHO grade glioma and among the total glioma samples (Fig. 7g). ChIP assays also showed that the enrichment of SRSF1 was decreased in HOXB5-silenced GSC406, while increased in HOXB5-overexpressed GSC201 (Fig. 7o). Finally, qRT-PCR and western blotting showed that SRSF1 expression was downregulated in HOXB5-silenced GSC406, while upregulated in HOXB5-overexpressed GSC201 (Fig. 7p, q, r). At the same time, we obtained the same results in U87 cells (Fig. S6f-i). Together, these results suggested that HOXB5 transcriptionally regulates SRSF1 expression in GSCs*.*

### The SRSF1/circATP5B/miR-185-5p/HOXB5 feedback loop regulates glioma tumorigenesis in vivo

**Fig. 8:**
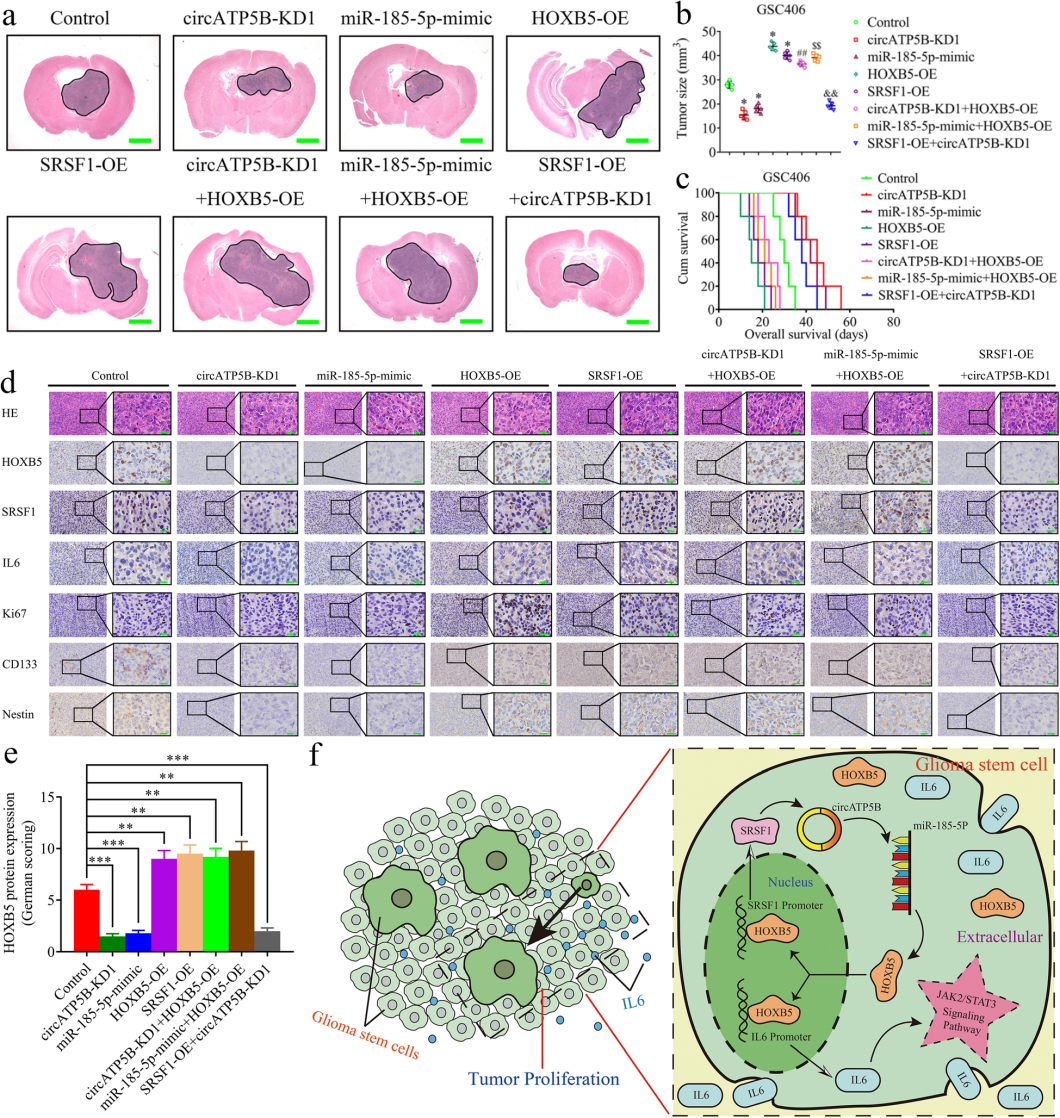
The SRSF1/circATP5B/miR-185-5p/HOXB5 feedback loop regulated glioma tumorigenesis in vivo. **a** Representative images showed the size of intracranial tumors in the coronal location of eight groups (negative control, circATP5B knockdown, miR-185-5p mimic, HOXB5 overexpression, SRSF1 overexpression, circATP5B knockdown combined with HOXB5 overexpression, miR-185-5p mimic combined with HOXB5 overexpression, SRSF1 overexpression combined with circATP5B knockdown in GSC406). Scale bar = 10 mm. **b** The measured tumor volumes among eight GSC406 groups are indicated. (*p < 0.05 vs. the negative control group; ^##^*p* < 0.05 vs. the circATP5B knockdown group, ^$$^*p* < 0.05 vs. the miR-185-5p group, ^&&^*p* < 0.05 vs. the SRSF1 overexpression group; Student’s t-test). **c** Kaplan-Meier survival curves showed that HOXB5 overexpression, SRSF1 overexpression, circATP5B knockdown combined with HOXB5 overexpression, miR-185-5p mimic combined with HOXB5 overexpression in GSC406 shortened the survival times of nude mice. At the same time, it prolonged the survival times after miR-185-5p mimic was transfected, circATP5B knockdown, and SRSF1 overexpression combined with circATP5B knockdown in GSC406. For each group, *n* = 5. **d** Representative immunohistochemical staining showing the changes in HOXB5, SRSF1, IL6, Ki67, CD133 and nestin in the negative control, circATP5B knockdown, miR-185-5p mimic, HOXB5 overexpression, SRSF1 overexpression, circATP5B knockdown combined with HOXB5 overexpression, miR-185-5p mimic combined with HOXB5 overexpression, SRSF1 overexpression combined with circATP5B knockdown group in orthotopic xenograft models. Scale bar = 50 μm. **e** The German scoring of HOXB5 protein expression in eight groups. (the circATP5B knockdown group vs. the negative control group: *p* < 0.001; the miR-185-5p mimic group vs. the negative control group: *p* < 0.001; the HOXB5 overexpression group vs. the negative control group: *p* < 0.01; the SRSF1 overexpression group vs. the negative control group: *p* < 0.01; the circATP5B knockdown combined with HOXB5 overexpression group vs. the negative control group: *p* < 0.01; the miR-185-5p mimic combined with HOXB5 overexpression group: *p* < 0.01; the SRSF1 overexpression combined with circATP5B knockdown group vs. the negative control group: *p* < 0.001; Student’s t-test). **f** Schematic diagram showing that the SRSF1/circATP5B/miR-185-5p/HOXB5 axis promoted glioma proliferation through IL6-mediated JAK2/STAT3 signaling pathway. All data were expressed as the mean ± SD (three independent experiments). EV: empty vector, OE: overexpression, NC: negative control, KD: knockdown. All data were expressed as the mean ± SD (three independent experiments). *p < 0.05; **p < 0.01; ***p < 0.001

We performed orthotopic xenografts to confirm the effects of the SRSF1/circATP5B/miR-185-5p/HOXB5 axis in glioma tumorigenesis in vivo. Compared with the control group, tumor volumes were decreased in the circATP5B knockdown group, the miR-185-5p mimic group, and the SRSF1 overexpression combined with circATP5B knockdown group. In contrast, tumor volumes were increased in the HOXB5 overexpression group, the SRSF1 overexpression group, the circATP5B knockdown combined with the HOXB5 overexpression group, and the miR-185-5p mimic combined with the HOXB5 overexpression group (Fig. 8a, b). Kaplan–Meier survival analysis showed similar results with the circATP5B knockdown group, the miR-185-5p mimic group, and the SRSF1 overexpression combined with circATP5B knockdown group showing longer median survival times compared with the normal control group. The opposite results were obtained in the HOXB5 overexpression group, the SRSF1 overexpression group, the circATP5B knockdown combined with HOXB5 overexpression group, and the miR-185-5p mimic combined with the HOXB5 overexpression group (Fig. 8c). Immunohistochemistry was performed to detect the effects of the SRSF1/circATP5B/miR-185-5p/HOXB5 axis on tumor tissues. The results confirmed that the circATP5B knockdown group, the miR-185-5p mimic group, and the SRSF1 overexpression combined with circATP5B knockdown group had lower expression of HOXB5, SRSF1, IL6, Ki67, CD133, and nestin, whereas higher expression of HOXB5, SRSF1, IL6, Ki67, CD133, and nestin was found in the HOXB5 overexpression group, the SRSF1 overexpression group, the circATP5B knockdown combined with HOXB5 overexpression group, and the miR-185-5p mimic combined with the HOXB5 overexpression group (Fig. 8d, e). To illustrate our findings, the schematic diagram in Fig. 8f shows that the SRSF1/circATP5B/miR-185-5p/HOXB5 feedback loop promotes the tumorigenesis and proliferation of glioma stem cells through the IL6-mediated JAK2/STAT3 signaling pathway. In summary, our results suggested that the SRSF1/circATP5B/miR-185-5p/HOXB5 axis regulates glioma tumorigenesis and proliferation in vivo.

## Discussion

CircRNAs are a type of highly stable and abundant endogenous non-coding RNA formed in the process of RNA splicing. Recently, an increasing number of circRNAs have been confirmed to regulate the development and progression of various human cancers, including breast cancer, hepatocellular carcinoma, and glioma [10, 39–41]. Several circRNAs are involved in the biological processes of glioma; however, little is known about the role of the novel circRNA, circATP5B, in glioma development and progression. This study found that circATP5B expression was significantly upregulated in clinical glioma specimens and patient-derived primary GSCs. According to Kaplan- Meier survival analyses, the higher expression of circATP5B correlated with a poorer prognosis in glioma patients, especially in higher WHO grade glioma patients. The capacity for active proliferation is regarded as a crucial feature of glioma, which correlates with patients’ poor prognosis [10]. Functionally, circATP5B promoted the proliferation of GSCs according to MTS, EDU, neurosphere formation, and limiting dilution assays, which implied that circATP5B is a tumor promoter in glioma. CircATP5B has potential as an efficient diagnostic biomarker and therapeutic target for glioma.

CircRNAs have been reported to have diverse molecular mechanisms in the development and progression of various cancers, among which competitive endogenous RNAs are the most frequently reported [11, 42]. CircRNAs contain one or more MREs that act as miRNA sponges to regulate miRNA-targeted genes [43, 44]. For example, circPTN sponges miR-145-5p/miR-330-5p to promote proliferation and stemness in glioma [10]. CircHIPK3 serves as a prognostic marker to promote glioma progression by regulating miR-654/IGF2BP3 signaling [34]. CircRNA hsa-circ-0014359 promotes glioma progression by regulating miR-153/PI3K signaling [2]. In our study, we confirmed that both circATP5B and HOXB5 possessed miR-185-5p binding sites, which implied the formation of a circATP5B/miR-185-5p/HOXB5 axis. We first confirmed the direct interaction between circATP5B and miR-185-5p and found that circATP5B negatively regulated miR-185-5p expression according to qRT-PCR, luciferase reporter assays, RIP assays, and Pearson’s correlation analyses. Functionally, we also confirmed that circATP5B promoted the proliferation of GSCs and that this effect was reversed by miR-185-5p, which suggested that circATP5B promoted the proliferation of GSCs by acting as a miR-185-5p sponge. Previous studies also demonstrated that miR-185-5p was downregulated in glioma, and played an inhibitory role in the development and progression of glioma [22, 45].

HOXB5 is a transcription factor that is overexpressed in several cancers and participates in the proliferation, migration, and invasion of cancer cells [14–16]. In our study, we certified that HOXB5 was overexpressed in clinical glioma specimens and patient-derived primary GSCs, and the higher expression of HOXB5 correlated with a poorer prognosis in glioma patients, as confirmed by qRT-PCR, western blotting, and Kaplan–Meier survival analyses. Functionally, HOXB5 was also confirmed to promote the proliferation of GSCs. Furthermore, miR-185-5p was shown to bind to the 3’UTR of HOXB5 mRNA, according to luciferase reporter assays, and negatively regulated the expression of HOXB5. We also confirmed that miR-185-5p suppressed the proliferation of GSCs, and that these suppressor effects were reversed by HOXB5, as demonstrated by MTS, EDU, and limiting dilution assays.

We further investigated the relationship between circATP5B and HOXB5 and confirmed that circATP5B actively upregulated HOXB5 expression, as confirmed by qRT-PCR and western blotting. This upregulation could be reversed by treatment with miR-185-5p mimic. Functionally, we also certified that circATP5B promoted the proliferation of GSCs by upregulating HOXB5 expression. Therefore, it is suggested that the circATP5B/miR-185-5p/HOXB5 axis is involved in the proliferation of GSCs.

We further studied the possible downstream effects of HOXB5 and confirmed that higher HOXB5 expression was associated with enrichment of IL6-mediated JAK2/STAT3 signaling. Interleukin-6 (IL-6), a pleiotropic proinflammatory cytokine, has been confirmed to play crucial roles in a wide range of biological activities, including immune regulation, inflammation, and oncogenesis through the activation of JAK2/STAT3 signaling [46]. Increasing evidence shows that the activation of IL6-mediated JAK2/STAT3 signaling is frequently associated with glioma, and promotes the cell growth, proliferation, and invasion of glioma cells [46–48]. In our study, we confirmed that HOXB5 transcriptionally regulated IL6 expression via qRT-PCR and western blotting, as well as ELISA, luciferase reporter, and ChIP assays. We also confirmed significantly positive correlations between HOXB5 and IL6 expression in each WHO grade glioma and among the total glioma samples. In addition, we showed that HOXB5 promoted the expression of downstream molecules of the JAK2/STAT3 signaling pathway by western blotting. Functionally, we confirmed that HOXB5 promoted the proliferation of GSCs via activating IL6-mediated JAK2/STAT3 signaling.

RBPs are involved in the post-transcriptional regulation of RNAs, as well as gene transcription and translation. They also play vital roles in both physiological and pathological processes [49]. RBPs have also been reported to participate in circRNA splicing and expression and promote the production of circRNAs [50, 51]. SRSF1, a splicing factor and a type of RBP, has been reported to be overexpressed in several cancers including glioma, and participates in diverse biological functions, including translation, nonsense-mediated RNA decay, and RNA transport [23–25]. In our study, we confirmed that SRSF1 bound to circATP5B and promoted circATP5B expression in GSCs via qRT-PCR, RIP and RNA pull-down assays. SRSF1 did not affect the expression of the ATP5B linear form. Functionally, we confirmed that SRSF1 promoted the proliferation of GSCs by binding to circATP5B, and synergetic effects were detected between SRSF1 and circATP5B in the proliferation of GSCs through MTS, EDU, and limiting dilution assays.

As a transcriptional factor, our study found two binding sites for HOXB5 in the promoter of SRSF1 and confirmed that HOXB5 transcriptionally regulated SRSF1 expression in GSCs*.* We also certified that there were significant positive correlations between HOXB5 and SRSF1 expression in clinical glioma specimens. Finally, we conclude that the SRSF1/circATP5B/miR-185-5p/HOXB5 feedback loop is involved in glioma tumorigenesis and proliferation.

## Conclusions

In summary, circATP5B was upregulated in glioma and this correlated with poor patient survival. We confirmed that circATP5B promoted the proliferation of glioma using patient-derived GSCs. Furthermore, HOXB5 transcriptionally regulated IL6 expression, and promoted the proliferation of GSCs via IL6-mediated JAK2/STAT3 signaling. Mechanistically, circATP5B upregulated the expression of HOXB5 in GSCs via miR-185-5p sponging. In addition, SRSF1 bound to circATP5B and promoted circATP5B expression, while HOXB5 also transcriptionally regulated and promoted SRSF1 expression in GSCs. Therefore, the SRSF1/circATP5B/miR-185-5p/HOXB5 feedback loop is considered to be involved in glioma proliferation. Our study has identified a novel potential biomarker for glioma diagnosis and prognosis evaluation, and may also offer a new target for glioma treatment.

## Supplementary Information

**Additional file 1: Supplementary Figure 1.** **a** Hematoxylin and eosin staining of the original patient tissues. Scale bar = 50 μm. **b** Immunofluorescence staining of CD133 and nestin in patient-derived GSCs. Scale bar = 50 μm. **c** Representative images showing that GSCs were differentiated and adherent (above). Immunofluorescence showing differentiated GSCs expressing GFAP or βIII tubulin (middle and below). Scale bar = 50 μm. **d** The quantifications of CD133 and nestin at the end of the incubation times as detected by western blotting. **e** qRT-PCR showed the expression of circATP5B in different patient-derived GSCs. (GSC302, GSC305 vs. GSC201, GSC203: *p* < 0.01; GSC403, GSC406 vs. GSC302, GSC305: *p* < 0.01; GSC403, GSC406 vs. GSC201, GSC203: *p* < 0.001; One-Way ANOVA). **f**, **g** The expression of HOXB5 in different patient-derived GSCs as detected by qRT-PCR (f) and western blotting (g). (qRT-PCR: GSC302, GSC305 vs. GSC201, GSC203: *p* < 0.01; GSC403, GSC406 vs. GSC302, GSC305: *p* < 0.01; GSC403, GSC406 vs. GSC201, GSC203: *p* < 0.001; One-Way ANOVA). **h**, **i** The expression of HOXB5 in different patient-derived GSCs and non-GSCs as detected by western blotting (h) and qRT-PCR (i). (qRT-PCR: GSC201 vs. nGSC201: *p* < 0.05; GSC305 vs. nGSC305: *p* < 0.01; GSC406 vs. nGSC406: *p* < 0.01; Student’s t-test). **j** The expression of circATP5B in glioma stem cells with different passage times as detected by qRT-PCR. (Passage 2,4,6,8 vs. passage 0: *p*>0.05; passage 10 vs. passage 0: *p* < 0.05; passage 12 vs. passage 0: *p* < 0.01; passage 14 vs. passage 0: *p* < 0.001; One-Way ANOVA). All data were expressed as the mean ± SD (three independent experiments). *p < 0.05; **p < 0.01; ***p < 0.001.

**Additional file 2: Supplementary Figure 2.** The expression of circATP5B, HOXB5, and SRSF1 in GSCs after lentiviral-based transfection. **a**, **b** The relative expression of circATP5B after circATP5B knockdown (a) or overexpression (b), as detected by qRT-PCR. ((a): GSC406: *p* < 0.01; One-Way ANOVA; (b): GSC201: *p* < 0.01; Student’s t-test). **c**, **d** The relative expression of HOXB5 after HOXB5 knockdown (c) or overexpression (d), as detected by qRT-PCR. ((c): GSC406: *p* < 0.001; One-Way ANOVA; (d): GSC201: *p* < 0.001; Student’s t-test). **e**, **f** The protein expression of HOXB5 after HOXB5 knockdown (e) or overexpression (f), as detected by western blotting. **g**, **h** The relative expression of SRSF1 after SRSF1 knockdown (g) or overexpression (h), as detected by qRT-PCR. ((g): GSC406: *p* < 0.01; One-Way ANOVA; (h): GSC201: *p* < 0.01; Student’s t-test). **i**, **j** The protein expression of SRSF1 after SRSF1 knockdown (i) or overexpression (j), as detected by western blotting. All data were expressed as the mean ± SD (three independent experiments). EV: empty vector, OE: overexpression, NC: negative control, KD: knockdown. *p < 0.05; **p < 0.01; ***p < 0.001.

**Additional file 3: Supplementary Figure 3.** CircATP5B promoted the proliferation of U87 cells through miR-185-5p sponging. **a**-**d** qRT-PCR showed the relative expression of circATP5B in U87 cells after miR-185-5p mimic (a, b) or inhibitor treatment (c, d). ((a, b): miR-185-5p mimic vs. miR-185-5p NC: *p* < 0.001; (c, d): miR-185-5p inhibitor vs. miR-185-5p NC: *p* < 0.001; Student’s t-test). **e**-**h** qRT-PCR showed the relative expression of miR-185-5p in U87 cells following circATP5B knockdown (e, f) or overexpression (g, h). ((e, f): *p* < 0.001; One-Way ANOVA; (g, h): *p* < 0.001; Student’s t-test). **i**, **j** MTS assays showed that circATP5B knockdown (i) or overexpression (j) affected the cell viability of U87 cells and was reversed by miR-185-5p inhibitor or mimic treatment, respectively. ((i): *p* < 0.001; One-Way ANOVA; (j): *p* < 0.001; One-Way ANOVA). **k**, **l** The EDU assays showed that circATP5B knockdown or overexpression affected the proliferation of U87 cells and was reversed by miR-185-5p inhibitor or mimic treatment, respectively. Scale bar = 100 μm. (U87: *p* < 0.01; One-Way ANOVA). EV: empty vector, OE: overexpression, NC: negative control, KD: knockdown. All data were expressed as the mean ± SD (three independent experiments). *p < 0.05; **p < 0.01; ***p < 0.001.

**Additional file 4: Supplementary Figure 4.** CircATP5B promoted the proliferation of U87 cells by upregulating HOXB5 expression. **a**-**d** qRT-PCR (a, b) and western blotting (c, d) showed the expression of HOXB5 in U87 cells after circATP5B knockdown or overexpression. (circATP5B-KD vs. circATP5B-NC: *p* < 0.01; circATP5B-OE vs. circATP5B-EV: *p* < 0.001; One-Way ANOVA). **e**, **f** The effects of both circATP5B and miR-185-5p on the expression of HOXB5 in U87 cells were detected by qRT-PCR. ((e): U87: *p* < 0.01; (f): U87: *p* < 0.01; One-Way ANOVA). **g** The decreased expression of HOXB5 in U87 cells induced by circATP5B knockdown was reversed by miR-185-5p inhibitor treatment, as detected by western blotting. **h** The increased expression of HOXB5 in U87 cells induced by circATP5B overexpression was reversed by miR-185-5p mimic treatment, as determined by western blotting. **i**, **j** MTS assays showed that circATP5B knockdown (i) or overexpression (j) affected the cell viability of U87 cells and was reversed by HOXB5 overexpression or knockdown, respectively. ((i): U87: *p* < 0.001; (j): U87: *p* < 0.001; One-Way ANOVA). **k**, **l** The EDU assays showed that circATP5B knockdown or overexpression affected the proliferation of U87 cells and was reversed by HOXB5 overexpression or knockdown, respectively. Scale bar = 100 μm. (U87: *p* < 0.01; One-Way ANOVA). EV: empty vector, OE: overexpression, NC: negative control, KD: knockdown. All data were expressed as the mean ± SD (three independent experiments). *p < 0.05; **p < 0.01; ***p < 0.001.

**Additional file 5: Supplementary Figure 5.** HOXB5 transcriptionally regulated IL6 expression and promoted the proliferation of U87 cells via IL6-mediated JAK2/STAT3 signaling. **a**, **b** qRT-PCR showed the IL6 expression was altered after HOXB5 knockdown or overexpression in U87 cells. (HOXB5-KD vs. HOXB5-NC: *p* < 0.01; HOXB5-OE vs. HOXB5-EV: *p* < 0.001; One-Way ANOVA). **c**, **d** MTS assays showed that HOXB5 knockdown (c) or overexpression (d) affected the cell viability of U87 cells and was reversed by additional recombinant IL6 or anti-IL6, respectively. ((c): U87: *p* < 0.001; (d): U87: *p* < 0.001; One-Way ANOVA). **e** The EDU assays showed that HOXB5 knockdown or overexpression affected the proliferation of U87 cells and was reversed by additional recombinant IL6 or anti-IL6, respectively. Scale bar = 100 μm. (U87: *p* < 0.01; One-Way ANOVA). EV: empty vector, OE: overexpression, NC: negative control, KD: knockdown. All data were expressed as the mean ± SD (three independent experiments). *p < 0.05; **p < 0.01; ***p < 0.001.

**Additional file 6: Supplementary Figure 6.** SRSF1 regulated the proliferation of U87 cells via upregulating circATP5B expression. **a**, **b** The relative expression of circATP5B after SRSF1 knockdown or overexpression was detected by qRT-PCR. (SRSF1-KD vs. SRSF1-NC: *p* < 0.01; SRSF1-OE vs. SRSF1-EV: *p* < 0.05; One-Way ANOVA). **c**, **d** MTS assays showed that SRSF1 knockdown (c) or overexpression (d) affected the cell viability of U87 cells and was reversed by circATP5B overexpression or knockdown, respectively. ((c): U87: *p* < 0.001; (d): U87: *p* < 0.001; One-Way ANOVA). **e** The EDU assays showed that SRSF1 knockdown or overexpression affected the proliferation of U87 cells and was reversed by circATP5B overexpression or knockdown, respectively. Scale bar = 100 μm. (U87: *p* < 0.01; One-Way ANOVA). **f**, **g** qRT-PCR showed the SRSF1 expression was affected after HOXB5 knockdown or overexpression in U87 cells. (HOXB5-KD vs. HOXB5-NC: *p* < 0.01; HOXB5-OE vs. HOXB5-EV: *p* < 0.001; One-Way ANOVA). **h**, **i** Western blotting showed the SRSF1 expression was affected after HOXB5 knockdown (h) or overexpression (i) in U87 cells. EV: empty vector, OE: overexpression, NC: negative control, KD: knockdown. All data were expressed as the mean ± SD (three independent experiments). *p < 0.05; **p < 0.01; ***p < 0.001.

**Additional file 7: Supplementary Table 1.** Clinical information of the primary glioma stem-like cells.

**Additional file 8: Supplementary Table 2.** siRNA sequences.

**Additional file 9: Supplementary Table 3.** PCR Primers.

## Data Availability

The datasets obtained and analyzed during the current study were made available from the corresponding authors through request.

## Abbreviations

GSCs: Glioma stem cells
circRNA: Circular RNA
HOX: Homeobox
miRNAs: MicroRNAs
RBPs: RNA-binding proteins
IL6: Interleukin-6
GSEA: Gene set enrichment analysis
TCGA: The Cancer Genome Atlas
CGGA: Chinese Glioma Genome Atlas
qRT-PCR: Real-Time Quantitative Reverse Transcription PCR
IHC: Immunohistochemistry
ELISA: Enzyme-linked immunosorbent assay
RIP: RNA immunoprecipitation
ChIP: Chromatin immunoprecipitation
RISC: RNA-induced silencing complex
ceRNAs: Competitive endogenous RNAs
LGG: Lower-grade glioma
GBM: Glioblastoma
