# Supplementary material for: The SRSF1/circATP5B/miR-185-5p/HOXB5 feedback loop regulates the proliferation of glioma stem cells via the IL6-mediated JAK2/STAT3 signaling pathway
Source: J Exp Clin Cancer Res. 2021 Apr 15;40:134. doi: 10.1186/s13046-021-01931-9 (PMC8051130; doi:10.1186/s13046-021-01931-9)
Supplement: Supplementary file 7 — Additional file 7: Supplementary Table 1. Clinical information of the primary glioma stem-like cells. [file 13046_2021_1931_MOESM7_ESM.docx]

Supplementary Table 1. Clinical information of the primary glioma stem-like cells

|  | GSC201 | GSC203 | GSC302 | GSC305 | GSC403 | GSC406 |
| --- | --- | --- | --- | --- | --- | --- |
| Gender | Female | Male | Male | Female | Female | Male |
| Age | 60 years old | 52 years old | 45 years old | 65 years old | 56 years old | 60 years old |
| Location | Right frontal lobe | Left insula | Left frontal lobe | Right parietal lobe | Right occipital lobe | Right occipital lobe |
| Pathological diagnosis | Astrocytoma | Astrocytoma | Anaplastic astrocytoma | Anaplastic astrocytoma | Glioblastoma | Glioblastoma |
| WHO grade | II | II | III | III | IV | IV |
| Ki-67 | 25% (+) | 35% (+) | 55% (+) | 50% (+) | 60% (+) | 60% (+) |
| IDH status | Wild | Wild | Wild | Wild | Wild | Wild |
| **1p/19q status** | Non-codeletion | Non-codeletion | Non-codeletion | Non-codeletion | Non-codeletion | Non-codeletion |
| **H3F3A status** | Mutant | Mutant | Mutant | Mutant | Mutant | Mutant |
| **MGMT status** | Unmethylation | Unmethylation | Unmethylation | Unmethylation | Unmethylation | Unmethylation |
