# Supplementary material for: The SRSF1/circATP5B/miR-185-5p/HOXB5 feedback loop regulates the proliferation of glioma stem cells via the IL6-mediated JAK2/STAT3 signaling pathway
Source: J Exp Clin Cancer Res. 2021 Apr 15;40:134. doi: 10.1186/s13046-021-01931-9 (PMC8051130; doi:10.1186/s13046-021-01931-9)
Supplement: Supplementary file 8 — Additional file 8: Supplementary Table 2. siRNA sequences. [file 13046_2021_1931_MOESM8_ESM.docx]

| **Primer** | **Forward (5’-3’)** | **Reverse (5’-3’)** |
| --- | --- | --- |
| circATP5B-KD1 | AAAAAUGAAGCUUUUUGGGUU | CCCAAAAAGCUUCAUUUUUCU |
| circATP5B-KD2 | AUAUUCACCUGCCAAAAUCUG | GAUUUUGGCAGGUGAAUAUGA |
| HOXB5-KD1 | AUGUAUUAAUGAAUUAUAGCG | CUAUAAUUCAUUAAUACAUCA |
| HOXB5-KD2 | UCUAUUUCGGUGAAAUUGGCG | CCAAUUUCACCGAAAUAGACG |
| SRSF1-KD1  SRSF1-KD2  siRNA-NC | AAAGAAUACGUGUAUAACCUA  UCAAAGAAAAGAAUACGUGUA  UUCUUCGAAGGUGUCACGUTT | GGUUAUACACGUAUUCUUUUC  CACGUAUUCUUUUCUUUGACC  ACGUGACACCUUCGAAGAATT |

**Supplementary Table 2. siRNA sequences**
