# Supplementary material for: The SRSF1/circATP5B/miR-185-5p/HOXB5 feedback loop regulates the proliferation of glioma stem cells via the IL6-mediated JAK2/STAT3 signaling pathway
Source: J Exp Clin Cancer Res. 2021 Apr 15;40:134. doi: 10.1186/s13046-021-01931-9 (PMC8051130; doi:10.1186/s13046-021-01931-9)
Supplement: Supplementary file 9 — Additional file 9: Supplementary Table 3. PCR Primers. [file 13046_2021_1931_MOESM9_ESM.docx]

**Supplementary Table 3. PCR Primers**

**qRT-PCR Primers**

| **Primer** | **Forward (5’-3’)** | **Reverse (5’-3’)** |
| --- | --- | --- |
| circATP5B | GCTGCACAAGAGCCTTGATT | ATACCCAGGATGGCAATGAT |
| HOXB5 | CCGCAGGGAACAACGATTG | GCCGTATTTGTAGAACACGTCCT |
| IL6 | ACTCACCTCTTCAGAACGAATTG | CCATCTTTGGAAGGTTCAGGTTG |
| SRSF1 | AACTCCTTCTCGGGGCGTTAT | CATCCCATTGTAATTGTAGCCGT |
| GAPDH | GGAGCGAGATCCCTCCAAAAT | GGCTGTTGTCATACTTCTCATGG |
| β-actin | CATGTACGTTGCTATCCAGGC | CTCCTTAATGTCACGCACGAT |

**ChIP qRT-PCR Primers**

| **Primer** | **Forward (5’-3’)** | **Reverse (5’-3’)** |
| --- | --- | --- |
| IL6 | GTAGGAGCAAGACGCAAG | GGAATTATGAATGAACCAGGC |
| SRSF1 | TCAGGCATCCTAGAGTGT | GGCAGCATTACTATTCATTC |
